# Metformin inhibits pancreatic cancer metastasis caused by SMAD4 deficiency and consequent HNF4G upregulation

**DOI:** 10.1007/s13238-020-00760-4

**Published:** 2020-07-31

**Authors:** Chengcheng Wang, Taiping Zhang, Quan Liao, Menghua Dai, Junchao Guo, Xinyu Yang, Wen Tan, Dongxin Lin, Chen Wu, Yupei Zhao

**Affiliations:** 1 Department of General Surgery, Peking Union Medical College Hospital, Chinese Academy of Medical Sciences and Peking Union Medical College, Beijing, 100730 China; 2 Department of Etiology and Carcinogenesis, National Cancer Center/Cancer Hospital, Chinese Academy of Medical Sciences and Peking Union Medical College, Beijing, 100021 China; 3 Collaborative Innovation Center for Cancer Personalized Medicine, Nanjing Medical University, Nanjing, 211166 China; 4 Sun Yat-sen University Cancer Center, State Key Laboratory of Oncology in South China, Guangzhou, 510060 China; 5 CAMS Oxford Institute (COI), Chinese Academy of Medical Sciences, Beijing, 100730 China

**Keywords:** pancreatic cancer, HNF4G, SMAD4 deficiency, SMAD4-deficient PDAC, Metformin

## Abstract

Pancreatic ductal adenocarcinoma (PDAC) has poor prognosis due to limited therapeutic options. This study examines the roles of genome-wide association study identified PDAC-associated genes as therapeutic targets. We have identified *HNF4G* gene whose silencing most effectively repressed PDAC cell invasiveness. *HNF4G* overexpression is induced by the deficiency of transcriptional factor and tumor suppressor SMAD4. Increased HNF4G are correlated with SMAD4 deficiency in PDAC tumor samples and associated with metastasis and poor survival time in xenograft animal model and in patients with PDAC (log-rank *P* = 0.036; HR = 1.60, 95% CI = 1.03–2.47). We have found that Metformin suppresses HNF4G activity via AMPK-mediated phosphorylation-coupled ubiquitination degradation and inhibits *in vitro* invasion and *in vivo* metastasis of PDAC cells with SMAD4 deficiency. Furthermore, Metformin treatment significantly improve clinical outcomes and survival in patients with SMAD4-deficient PDAC (log-rank *P* = 0.022; HR = 0.31, 95% CI = 0.14–0.68) but not in patients with SMAD4-normal PDAC. Pathway analysis shows that HNF4G may act in PDAC through the cell-cell junction pathway. These results indicate that SMAD4 deficiency-induced overexpression of HNF4G plays a critical oncogenic role in PDAC progression and metastasis but may form a druggable target for Metformin treatment.

**Electronic supplementary material:**

The online version of this article (10.1007/s13238-020-00760-4) contains supplementary material, which is available to authorized users.

## INTRODUCTION

Pancreatic ductal adenocarcinoma (PDAC) is the seventh leading cause of cancer deaths worldwide (Bray et al., 2018; Ferlay et al., 2019). Globally, the incidence of pancreatic cancer is nearly the same as mortality and the five-year survival rate is about 8% (Siegel et al., 2018). A formidable challenge remains for PDAC therapy. Although surgery followed by adjuvant chemotherapy is the first-line therapy (Neoptolemos et al., 2018), such treatment has not yet resulted in a desired outcome for PDAC patients. The high lethality of this disease has driven to develop new therapeutic strategies. However, numerous target agents under evaluation have so far failed to significantly improve patient survival although Erlotinib and Olaparib have been shown to be of statistically significant, yet clinically marginal, benefit for the prognosis of PDAC patients (Moore et al., 2007; Golan et al., 2019). The failure of target therapies to pancreatic cancer might be attributable to high molecular heterogeneity of the cancer (Neoptolemos et al., 2018). In recent years, whole-exome or whole-genome sequencing studies have discovered many genomic alternations in PDAC (Bailey et al., 2016) and the results may help to develop future new precision therapies; however, addressing other molecular mechanisms for PDAC development and progression such as genetic variants and epigenetic modifications of genes are also needed for precision care of this malignancy.

Genome-wide association study (GWAS) is a powerful tool to identify genetic variants associated with risk and phenotypes of diseases. In the past 10 years, 8 GWAS on different racial populations have been published and identified 79 genetic variants associated with PDAC (Amundadottir et al., 2009; Low et al., 2010; Petersen et al., 2010; Wu et al., 2011; Li et al., 2012; Wolpin et al., 2014; Childs et al., 2015; Klein et al., 2018). However, the functions and the action mechanisms of these associated variants remain largely unknown. It has been proposed that some GWAS-identified disease risk variants may also contribute to the disease progression or even outcomes through their molecular functions on disease phenotypes including response to therapies. Indeed, we have previously demonstrated that a GWAS-identified PDAC risk variant *BACH1* is also associated with poor survival of patients, which may be mediated by resistance of the variant genotype to gemcitabine treatment (Huang et al., 2018). Another example is that we found a genetic variant in the 5′UTR of *SLC39A6* associated with shorter survival in esophageal cancer (Wu et al., 2013), which may be attributed to alleviating *SLC39A6* repression and promoting cancer cell invasion and metastasis (Cheng et al., 2017). Therefore, it is warranted to explore the potential roles of the genetic susceptibility variants in disease progression and in therapeutic targeting.

In this study, we have investigated the GWAS-identified 36 genes whose variants are associated with risk of PDAC by small interfering RNAs and found that the hepatic nuclear factor 4γ (*HNF4G*) was an important player promoting PDAC progression and invasiveness. HNF4G was previously identified as a transcription factor belonging to nuclear hormone receptor superfamily. Several studies have suggested that *HNF4G* might act as an oncogene modulating cell proliferation and invasion in cancer (Okegawa et al., 2013; Shukla et al., 2017; Wang et al., 2018). However, the function and action mechanism of HNF4G in pancreatic cancer remain unknown. Here, we demonstrate that SMAD4 deficiency upregulates HNF4G expression in PDAC, which can be suppressed by Metformin via AMPK-mediated phosphorylation-coupled ubiquitination degradation. Metformin treatment significantly represses progression and metastasis of transplanted PDAC in mice and improves the clinical outcomes in patients with SMAD4-deficient PDAC.

## RESULTS

### HNF4G is an important player in the progression and invasiveness of PDAC

**Figure 1:**
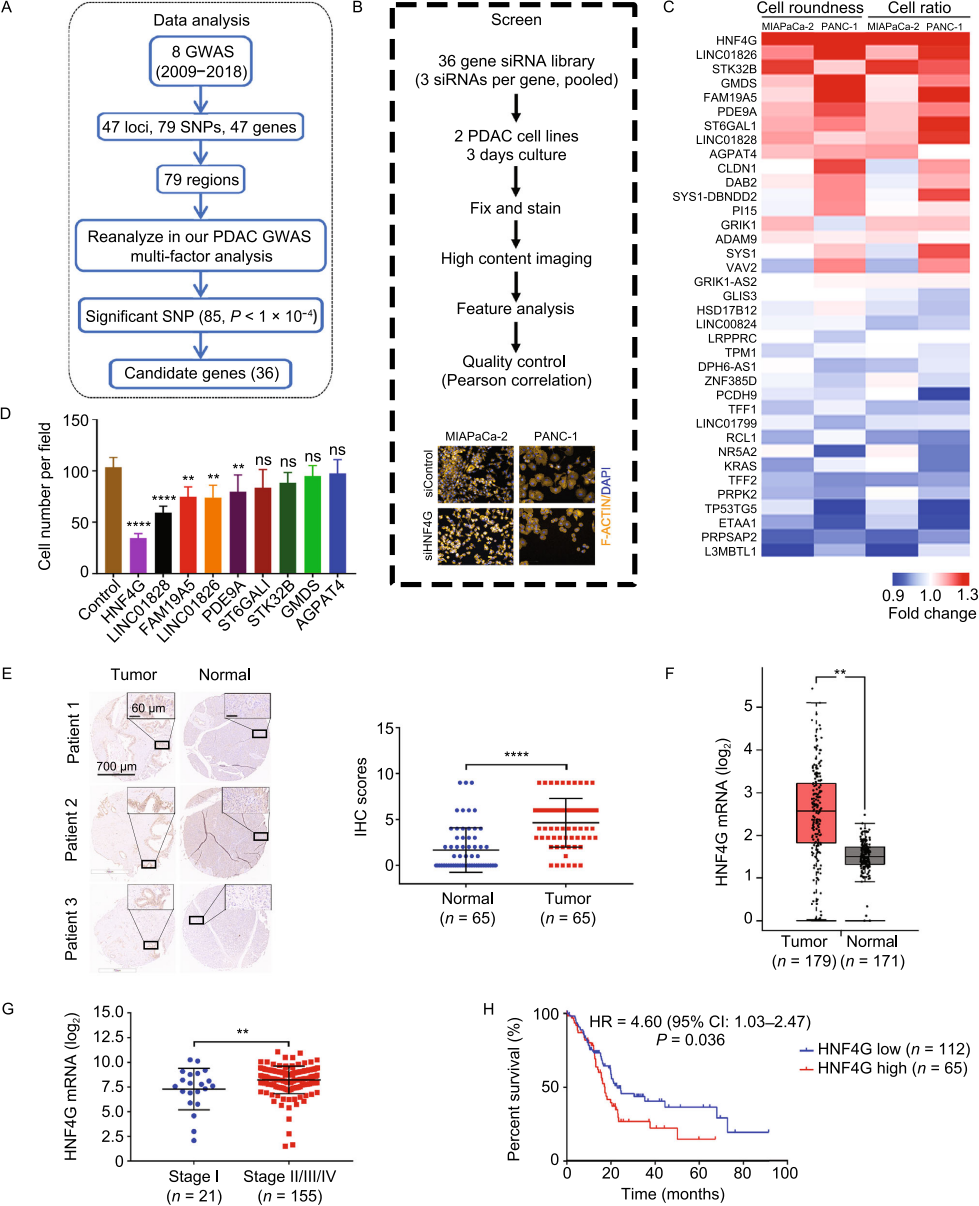
***HNF4G*** **is an important player in PDAC progression and invasiveness.** (A) Scheme of reanalyzing PDAC susceptibility genes using combined GWAS data. (B) High content screening strategy for 36 candidate genes in PDAC cells. Scale bar: 100 μm. (C) Heatmap showing the results of high content screening with a siRNAs library in PDAC cells. (D) The effect of siRNA knockdown of indicated genes on migration ability of PANC-1 cells. Data represent mean ± SEM from 3 experiments. (E) Immunohistochemical (IHC) staining of HNF4G in tissue array consisting of 65 PDAC samples. Left panel, representative IHC images, Scale bar: 700 μm (left images) and 60 μm (right images); right panel, quantification statistic. (F) Scatter dot plots showing HNF4G expression levels in PDAC tumor and normal samples. Data are derived from the Gene Expression Profiling Interactive Analysis (GEPIA). (G) Scatter dot plots showing HNF4G expression levels in early and latter stage of PDAC. Data were derived from the TCGA PDAC dataset. (H) Kaplan-Meier plots of overall survival of patients derived from the TCGA PDAC dataset stratified by HNF4G expression. The best performing threshold is used as a cutoff. HR, hazard ratio; CI, confidence interval. Statistical significance: *, *P* < 0.05, **, *P* < 0.01 and ****, *P* < 0.0001 of Student’s *t*-test or Wilcoxon rank-sum test. ns, not significant

By joint analysis and fine mapping of previously published 8 GWAS data (See Methods; Table S1), we discovered 36 genes significantly associated with PDAC (*P* < 1 × 10^−4^; Fig. 1A and Table S2). We then conducted high-throughput siRNA screening of these 36 genes to examine whether they have effects on PDAC cell phenotypes (Fig. 1B) and found that knocking down expression of 9 genes significantly repressed but 6 genes significantly promoted PDAC cells invasiveness indicated by cell roundness and ratio (Figs. 1C, S1A, S1B and Table S3). In this study, we focused on genes having potential oncogenic roles because they are more likely to be therapeutic targets. We then examined the effects of the 9 genes that have potential oncogenic role by using their individual siRNAs. Transwell assays showed that, among these genes, *HNF4G* was the most effective in repressing PDAC cell migration and invasion when its expression was silenced (Figs. 1D and S1C). IHC staining of tissue arrays showed that HNF4G expression levels were significantly higher in PDAC tumor than in adjacent normal tissues (*P* < 0.0001; Fig. 1E). Combined analysis of PDAC data in both The Cancer Genome Atlas (TCGA) and the genotype-tissue expression (GTEx) also showed significantly higher *HNF4G* RNA levels in tumor than in normal samples (*P* < 0.001; Fig. 1F). Furthermore, we found that the elevated *HNF4G* RNA levels were significantly correlated with advanced tumor stage (*P* = 0.008, Fig. 1G) and poor survival (HR = 1.60, 95% CI = 1.03–2.47; Fig. 1H). Collectively, these results strongly support that *HNF4G* is an important player in PDAC development and progression.

### HNF4G overexpression is associated with SMAD4 deficiency in PDAC

**Figure 2:**
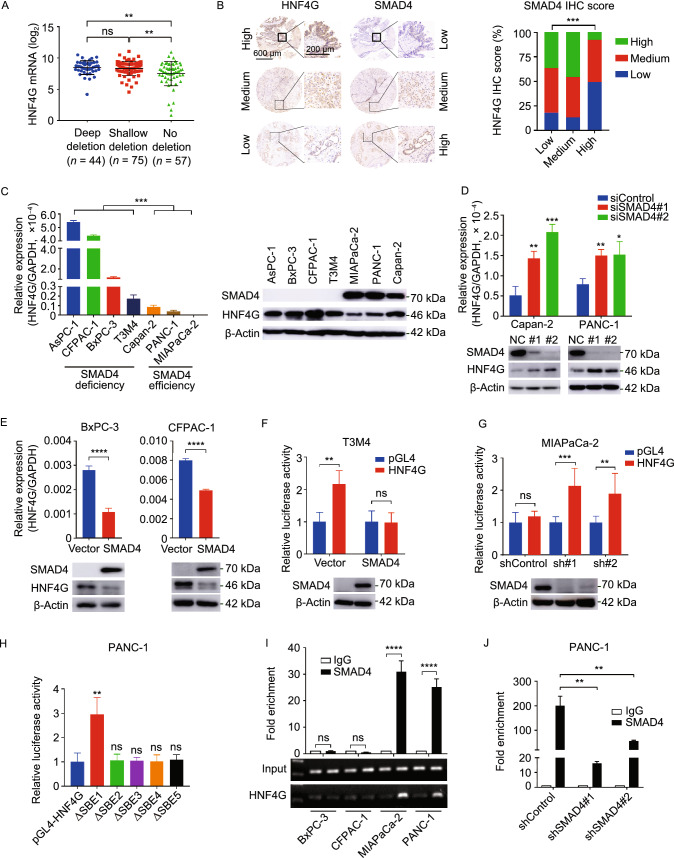
***HNF4G*** **upregulation is caused by** ***SMAD4*** **deficiency in PDAC.** (A) *HNF4G* mRNA levels in PDAC as function of *SMAD4* copy-number variation. Data were derived from the TCGA database. (B) The relationship between HNF4G and SMAD4 protein levels in PDAC determined by IHC staining. *Left panel* shows representative IHC images of HNF4G and SMAD4 in serial sections of PDAC tissue array (*n* = 185). Scale bar in left images = 600 μm. Scale bar in right images = 200 μm. Right panel shows HNF4G levels as function of SMAD4 levels both expressed as IHC scores: low, 0; medium, 1–4; and high, 6–12. (C) The expression levels of *HNF4G* RNA (left) and protein (right) in 4 PDAC cell lines with SMAD4 deficiency and 3 cell lines without SMAD4 deficiency. Data represent mean ± SEM from 3 independent determinations and each had triplicates. (D and E) The effects of *SMAD4* knockdown (D) or overexpression (E) on HNF4G RNA (upper panel) and protein levels (lower panel) in PDAC cells. Data are mean ± SEM from 3 independent determinations and each had triplicates. (F and G) Relative expression levels of reporter gene bearing the *HNF4G* promoter region in T3M4 cells with or without SMAD4 overexpression (F) and in MIAPaCa-2 cells with or without SMAD4 knockdown (G). (H) Relative expression levels of reporter gene bearing the mutated *HNF4G* promoter region in PDAC cells. Each promoter harbors a mutated SBE. Mutation in SBE 1 had the most significant impact on reporter gene expression compared with vector control and the mutation in other SBEs. Results are mean ± SEM from 3 experiments and each had 6 replicates. (I and J) Chromatin immunoprecipitation assays showing binding of SMAD4 to HNF4G promoter region SBE 1 in PDAC cells (I) and knockdown of SMAD4 expression in these cells substantially decreased the binding (J). Fold enrichment represents DNA levels associated with HNF4G or IgG (as control) relative to an input control from 3 independent experiments. Data are mean ± SEM of 3 experiments. Statistical significance: *, *P* < 0.05, **, *P* < 0.01, ***, *P* < 0.001 and ****, *P* < 0.0001 of Student’s *t*-test, χ^2^ test or Wilcoxon rank-sum test. ns, not significant

To seek for why *HNF4G* is overexpressed in PDAC, we looked at genomic alterations including SNP, CNV and methylation status of *HNF4G* in PDAC tissues and cell lines derived from different datasets but the results were negative (Fig. S2A and S2B). Nevertheless, analysis of *HNF4G* promoter and enhancer sequences using the GIAGEN software suggested 115 transcription factor binding sites (Table S4). Further analysis of TCGA data showed that PDAC tumors with deletion of *SMAD4*, a driver gene frequent loss-of-function in PDAC and a gene previously reported to regulate *HNF4G* expression in enterocyte (Chen et al., 2019), had higher *HNF4G* RNA levels than those without *SMAD4* deletion (*P* = 0.004; Fig. 2A). Furthermore, PDAC tumors with *SMAD4* truncation mutations also had higher *HNF4G* RNA levels compared with those without such mutations or with other mutation types (Fig. S2C). These results were confirmed in our PDAC samples analyzed by Immunohistochemical (IHC) staining showing that SMAD4 expression levels were inversely correlated with HNF4G levels (Fig. 2B). The inverse correlation between *SMAD4* and *HNF4G* expression levels were also seen in PDAC cell lines where the *SMAD4* gene deletion status was known (Fig. 2C). Additionally, we found that knockdown of *SMAD4* expression in PDAC cells, where *SMAD4* is normal, significantly increased *HNF4G* expression at both RNA and protein levels, while ectopic expression of *SMAD4* in PDAC cells, where *SMAD4* is deficient, significantly repressed *HNF4G* expression (Fig. 2D and 2E).

Next, we examined how SMAD4 regulates *HNF4G* expression. Bioinformatics indicated 5 SMAD binding elements (SBEs; GTCTG) and one stretch of transforming growth factor-β (TGF-β) inhibitory elements (TIE; GCCAAGC) within the *HNF4G* promoter region (Fig. S2D and S2G). These findings suggested that the canonical TGF-β signaling inhibits transcription of *HNF4G* gene through SMAD4. We thus conducted reporter gene assays with a plasmid construct carrying the −1,200 base pairs to +1,400 base pairs of *HNF4G* promoter sequences centered by transcriptional start site (TSS) predicted by Promoter 2.0 Prediction Server. The reporter expression levels were significantly decreased when *SMAD4* was overexpressed in T3M4 and BxPC-3 cells (Figs. 2F and S2E); however, the levels were significantly increased in MIAPaCa-2 and PANC-1 cells when *SMAD4* expression was knocked down (Figs. 2G and S2F). In addition, the reporter assays with mutated *HNF4G* promoter at each SBE (Fig. S2G) showed that only mutation at the 1st SBE dramatically increased reporter expression level compared with wild-type *HNF4G* promoter (Fig. 2H). ChIP-qPCR analysis showed a significant enrichment of *HNF4G* in cells with normal *SMAD4* but not in cells with deficient *SMAD4* (Fig. 2I). Knockdown of *SMAD4* expression in cells with normal *SMAD4* also significantly reduced the interaction between SMAD4 and *HNF4G* promoter (Figs. 2J and S2H). Together, these results demonstrate an inhibitory role of SMAD4 in *HNF4G* transcription.

### *HNF4G* activates the cell-cell junction pathway to promote PDAC metastasis

**Figure 3:**
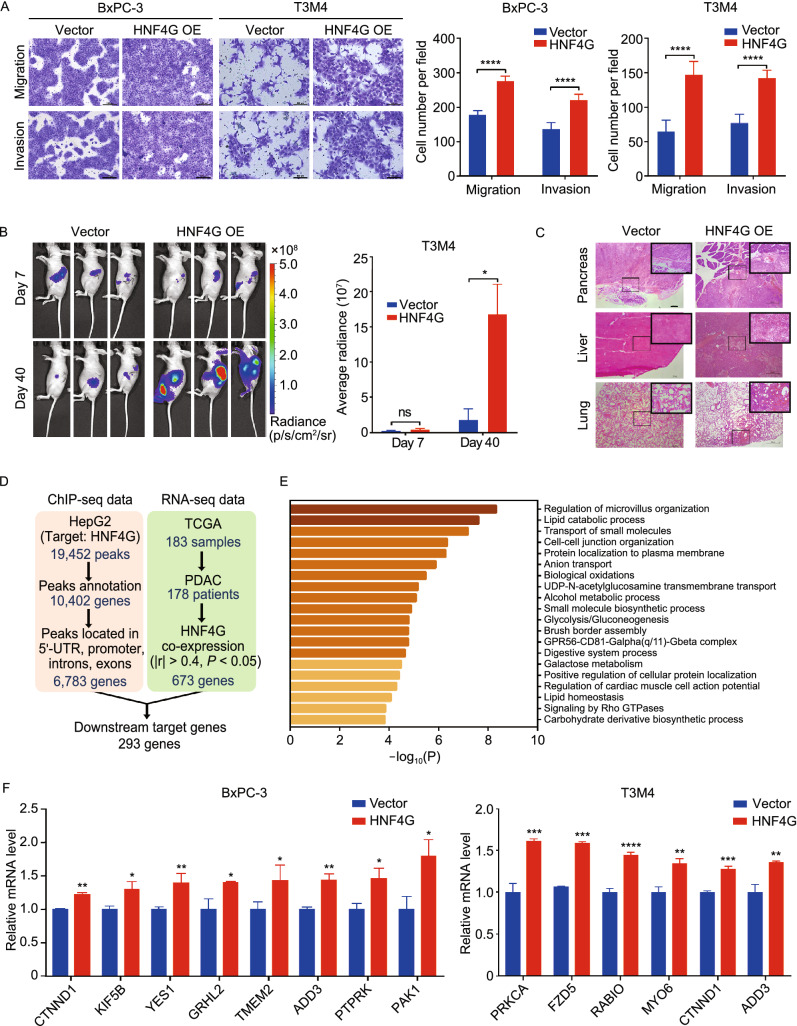
**HNF4G overexpression promotes PDAC cell invasiveness and activates the cell-cell junction pathway.** (A) HNF4G overexpression promoted migration and invasion of PDAC cells *in vitro*. Left panel shows representative images of transwell assays and right panel shows quantification statistic. Data are mean ± SEM from 3 independent experiments and each had duplicate. (B) HNF4G overexpression promoted migration and invasion of PDAC cells transplanted in the pancreas of mice (*n* = 3). Left panel shows representative bioluminescence images taken at 7 and 40 days of implantation; Right panel shows quantitative fluorescent intensity of the transplanted PDAC. (C) Representative H&E staining pictures of the pancreas, liver and lung from mice implanted orthotopically with PDAC cells with or without HNF4G overexpression. Scale bars: 100 μm. (D) The work flow schematic for analyzing the candidate genes targeted by HNF4G. (E) Functional enrichment of the 293 HNF4G-targeted genes by Metascape. (F) The expression levels of some downstream genes of HNF4G in PDAC cells with or without HNF4G overexpression. Results are mean ± SEM from 3 independent determinations and each had triplicate. Statistical significance: *, *P* < 0.05; **, *P* < 0.01; ***, *P* < 0.001 and ****, *P* < 0.0001 of Student’s *t*-test or Wilcoxon rank-sum test. ns, not significant

We then explore the effect of HNF4G on the capabilities of *in vitro* migration and invasion and *in vivo* metastasis in xenograft tumor models by stable overexpression or knockout of this gene in PDAC cells (Fig. S3A). We found that *HNF4G* knockout significantly suppressed (Fig. S3B) but *HNF4G* overexpression substantially enhanced the activities of cell migration and invasion *in vitro* (Fig. 3A). Similar results were obtained in mouse pancreatic orthotopic implantation of T3M4 cells, showing that *HNF4G* overexpression significantly promote metastasis of the xenografts to other organs such as liver, spleen and lung as measured by luminescence imaging (Fig. 3B) and confirmed by histopathology (Fig. 3C). These results suggest that HNF4G plays a pivotal role in PDAC metastasis.

Based on analyzing the chromatin immunoprecipitation sequencing (ChIP-seq) data in the Gene Expression Omnibus (GEO) database and the RNA-sequencing data in the TCGA PDAC database, we found 6,783 genes that might be regulated by HNF4G and among them, 293 genes were significantly co-expressed with *HNF4G* (Fig. 3D and Table S5). Of the 293 genes, 32 were enriched in the cell-cell junction pathway (Fig. 3E and Table S6), an important expression program relative to cell junction integrity and cancer metastasis (Runkle and Mu, 2013; Martin, 2014; De Pascalis and Etienne-Manneville, 2017). We found that in PDAC cells, the expressions of several genes in the cell-cell junction pathway were significantly altered by ectopic overexpression or knockdown of *HNF4G* (Figs. 3F and S3C), suggesting that the role of *HNF4G* overexpression in promoting PDAC metastasis may be through regulating the cell-cell junction pathway.

### Metformin activates AMPK that induces HNF4G phosphorylation-ubiquitination degradation

**Figure 4:**
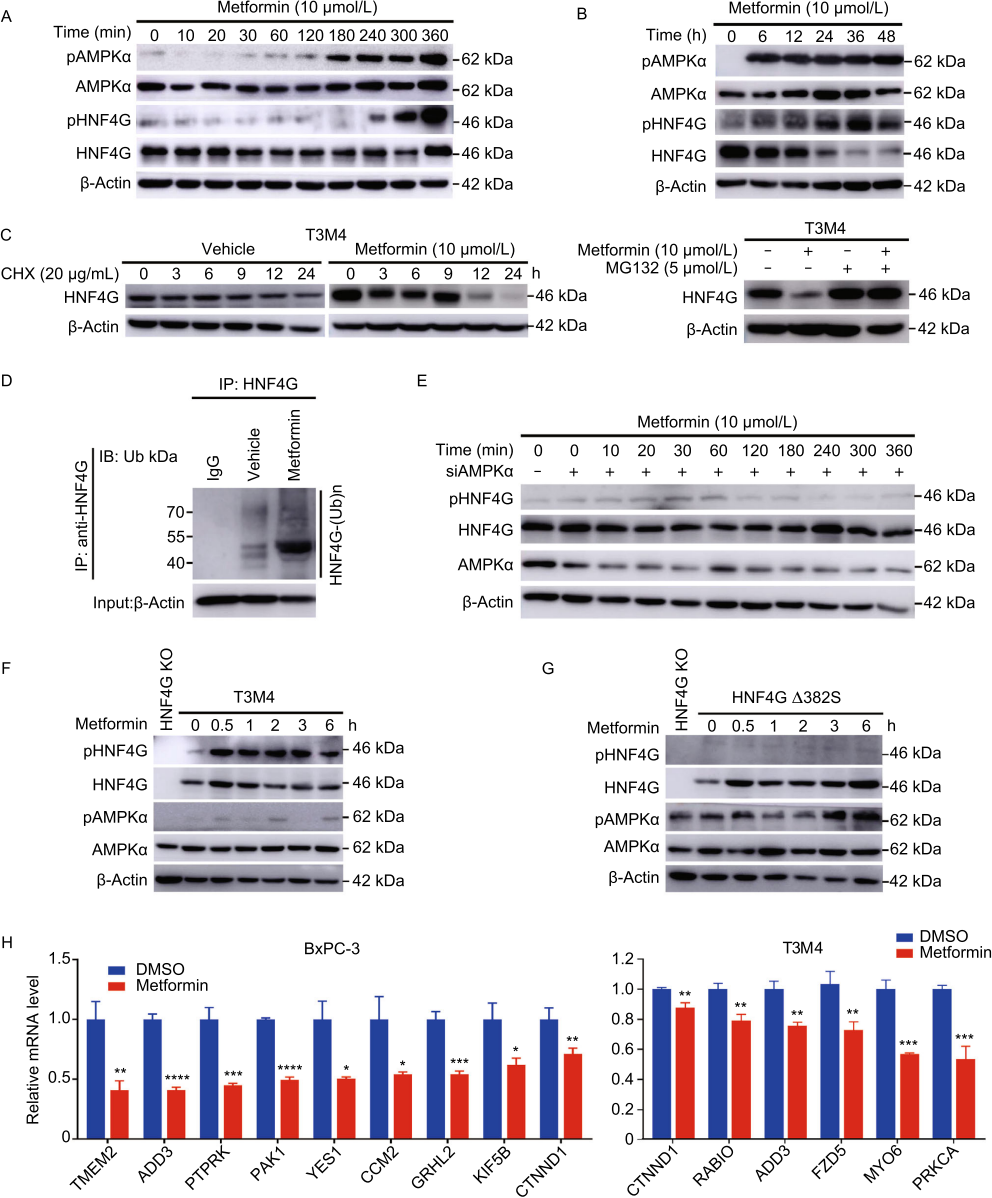
**Metformin activates AMPK that induces HNF4G phosphorylation-ubiquitination coupled degradation.** (A) Effect of Metformin (10 μmol/L) on HNF4G and AMPKα phosphorylation in T3M4 cells. (B) Metformin (10 μmol/L) promoted AMPKα phosphorylation and HNF4G degradation in T3M4 cells. (C) Metformin promoted HNF4G degradation but not inhibited its synthesis in T3M4 cells. Left panel, Metformin treatment substantially decreased the HNF4G levels with time in cells exposed to protein synthesis inhibitor cycloheximide (CHX; 20 μg/mL) compared with cells exposed to vehicle; right panel, Metformin treatment no longer substantially decreased HNF4G level in cells exposed to proteasome inhibitor MG132 (5 μmol/L). (D) Metformin promotes HNF4G ubiquitination. T3M4 cells were treated with or without Metformin (10 μmol/L). Cell lysates were immunoprecipitated (IP) with either control IgG or antibody against HNF4G and analyzed by immunoblotting with a ubiquitin (Ub)-specific antibody. Bottom panels, input from cell lysates. (E) Immunoblot analysis of HNF4G phosphorylation status in T3M4 cells with or without AMPKα knockdown treated with Metformin (10 μmol/L). (F and G) Immunoblot analysis of phosphorylated HNF4G and AMPKα in T3M4 cells, cells with HNF4G knockout or cells with ectopic expression of S382A-mutated HNF4G exposed to Metformin (10 μmol/L) for different times. (H) Metformin treatment significantly decreased the expression levels of some oncogenes in PDAC cells compared with vehicle controls. Results are mean ± SEM from 3 independent determinations and each had triplicate. Statistical significance: *, *P* < 0.05; **, *P* < 0.01; ***, *P* < 0.001; and ****, *P* < 0.0001 of Student’s *t*-test

Since HNF4G overexpression promotes PDAC metastasis, inhibiting its activity might be an option for PDAC therapy. By analyzing HNF4G protein sequences using the Scansite4, we found 29 motifs that could be phosphorylated by several kinases including PK3R1 and PRKAA1 (Table S7) for that the agonists are currently available. We treated PDAC cells with Isoprenaline (PIK3R1 agonist) or Metformin (PRKAA1 agonist) and found that Metformin but not Isoprenaline substantially enhanced HNF4G phosphorylation and consequent ubiquitination degradation (Figs. 4A–D and S4A–D). This effect was abolished when adenosine 5′-monophosphate (AMP)-activated protein kinase α (AMPKα) expression was knocked down in cells (Fig. 4E), indicating that Metformin-induced HNF4G phosphorylation and degradation was likely mediated by AMPKα. By analyzing HNF4G sequence, we identified 3 potential AMPK-phosphorylation sites including threonine 143 (T143), serine 161 (S161) and serine 382 (S382). Indeed, *in vitro* assays showed that the HNF4G-alanine 382 mutant (A382) had significantly reduced phosphorylation by AMPK compared with the HNF4G-serine 382 wild-type (S382), while the HNF4G-arginine 143 (R143) or HNF4G-alanine 161 (A161) mutant had little effect (Figs. 4F, 4G, S4E and S4F). Consistent with these results, motif analysis also showed a great similarity between the canonical AMPK substrate motif and the HNF4G S382 site that is highly conserved in different species (Fig. S4G). In addition, the expressions of HNF4G target genes were abrogated in HNF4G-overexpressing cells treated with Metformin (Fig. 4H). Collectively, these results indicate that the effect of Metformin on promoting phosphorylation/ubiquitination-coupled degradation of HNF4G is mediated by AMPK activation.

### Metformin suppress HNF4G-induced PDAC metastasis depending on SMAD4 status

**Figure 5:**
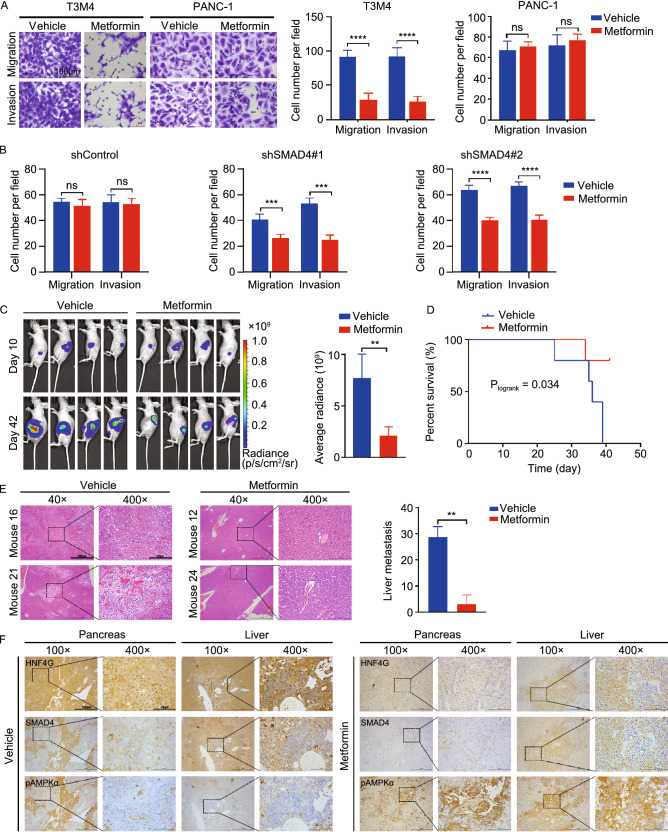
**Metformin suppress HNF4G-induced PDAC metastasis depending on SMAD4 status.** (A) Metformin treatment significantly repressed *in vitro* migration and invasion of SMAD4-deficient T3M4 cells but not SMAD4-efficient PANC-1. Left panels show representative images of transwell assays and right panels represent quantitative statistic. Data are mean ± SEM from 3 independent experiments and each had triplicate. Shown are the results in cells treated with or without Metformin (10 μmol/L); see also Fig. S5 for the entire and detailed dose-dependent results. (B) Knockdown of SMAD4 expression significantly promoted Metformin to suppress the migration and invasion of PDAC cells. Upper panel are representative transwell images and lower panel are quantitative data (mean ± SEM from 3 independent experiments and each had triplicate). (C) Metformin treatment significantly repressed the spread of HNF4G-overexpressing T3M4 cells implanted in mouse pancreas (*n* = 4). Left panel shows bioluminescence images of mice and the right panel shows quantitative fluorescent intensities. (D) Metformin treatment significantly prolonged survival time of mice implanted with PDAC in the pancreas as compared with vehicle control. (E) Metformin treatment significantly reduced PDAC metastases in the liver compared with vehicle control. Left panel shows representative tumor nodes staining by H&E and right panel shows quantitative statistic. Scale bars: 100 μm (40×) and 10 μm (400×). (F) Representative IHC pictures showing that Metformin treatment substantially reduced HNF4G but increased p-AMPKα expression levels in serial sections of both pancreas and liver from mice with PDAC implantation as compared with vehicle control. Scale bars: 100 μm (100×) and 25 μm (400×). Statistical significance: **, *P* < 0.01; ***, *P* < 0.001; and ****, *P* < 0.0001 of Student’s *t*-test or Wilcoxon rank-sum test. ns, not significant

We next examined whether Metformin suppresses cell invasiveness and metastasis induced by HNF4G overexpression. *In vitro* assays showed that Metformin significantly inhibited PDAC cell migration and invasion in a concentration-dependent manner; however, this effect was only seen in T3M4 and BxPC-3 cell lines that are known *SMAD4* loss-of-function mutants but not in PANC-1 cell line that is *SMAD4* wild-type (Figs. 5A and S5A). The dependency of Metformin action upon the *SMAD4* status was further verified in *SMAD4*-wild type cells with *SMAD4* knockdown by its shRNA (Figs. 5B and S5B). *In vivo* experiments showed that treatment with Metformin of mice carrying PDAC xenograft derived from HNF4G-overexpressing T3M4 cells in the pancreas significantly reduced the tumor burden and prolonged survival time compared with treatment with vehicle (Fig. 5C and 5D). Furthermore, histopathological analysis demonstrated that Metformin treatment significantly decreased metastatic tumor number in the liver (Fig. 5E). However, we did not observe significant differences in the body weight of mice treated with Metformin or vehicle (Fig. S5C), indicating that Metformin is not toxic at this effective dose. We then performed immunohistochemical analysis in serial sections of xenograft tumors and liver metastases and the results showed that treatment with Metformin substantially increased the p-AMPK levels but reduced the HNF4G levels as compared with treatment with vehicle (Fig. 5F). Collectively, both *in vitro* and *in vivo* results indicated that Metformin is an effective therapeutic agent targeting HNF4G for treatment of S*MAD4* deficient PDAC.

### Metformin treatment improves clinical outcomes in patients with SMAD4-deficient PDAC

**Figure 6:**
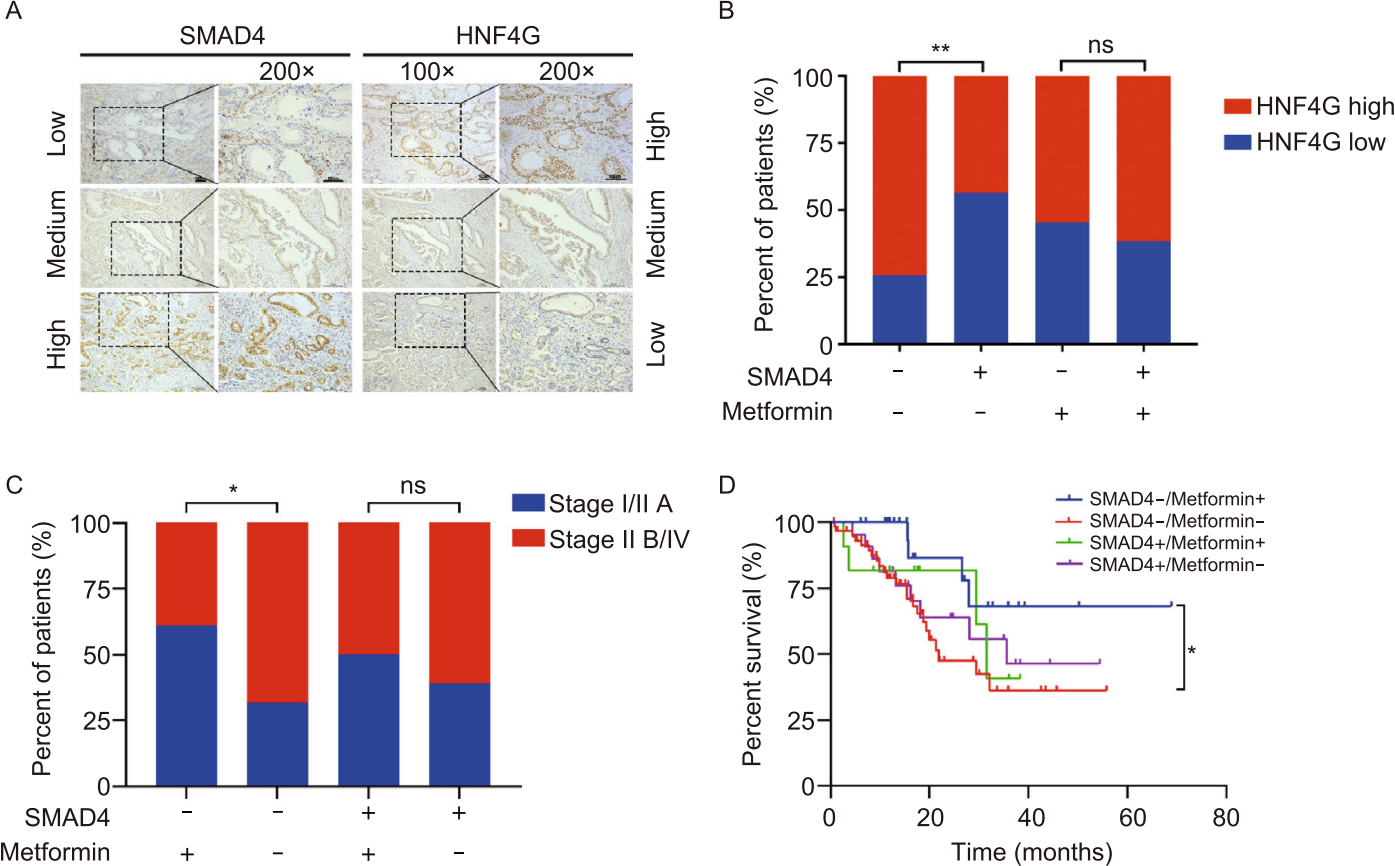
**Metformin treatment improves clinical outcomes of patients with SMAD4-deficient PDAC.** (A) Representative images of IHC staining of SMAD4 and HNF4G proteins in PDAC. Scale bar = 100 μm. (B) The correlation between HNF4G and SMAD4 protein levels in PDAC determined by IHC score. SMAD4−, IHC score = 0; SMAD4+, IHC score > 0. HNF4G Low level, scores 0–4; HNF4G high level, scores 6–12. (C) The distribution of patients by PDAC tumor stage and SMAD4 status as function of Metformin treatment. (D) Kaplan-Meier estimate of survival time in 118 patients with PDAC by SMAD4 status and Metformin treatment. Hazard ratio (HR) and 95% confidence interval (CI) were calculated with age, sex, tumor stage as covariates. SMAD4−, IHC score = 0; SMAD4+, IHC score > 0. Statistical significance: *, *P* < 0.05 and**, *P* < 0.01 of χ^2^ test. ns, not significant

To examine the curative effect of Metformin on PDAC, we recruited 139 individuals who concurrently suffered from type 2 diabetes mellitus and therefore were administrated with Metformin or other drugs prior to PDAC diagnosis (Table S8). IHC analysis of SMAD4 and HNF4G expression in this PDAC sample set (Fig. 6A) showed 74.1% (103/139) of SMAD4 deficiency. The significant inverse correlation between SMAD4 levels and HNF4G levels were confirmed in non-Metformin treatment group (*P* = 0.0012) but not in Metformin group (Fig. 6B) because HNF4G was substantially degraded in Metformin group. We analyzed the effect of Metformin treatment on clinical outcomes in patients who had complete tumor staging data (*n* = 113) or survival data (*n* = 118). We found that among patients with SMAD4-deficient PDAC, the proportion of low-staged tumors (stages IA, IB and IIA), classified upon diagnosis, was significantly higher in those with Metformin treatment than that in those without Metformin treatment (60.9% versus 31.7%, *P* = 0.015); however, this significant difference was not seen in patients with SMAD4-normal PDAC (Fig. 6C). More importantly, we found that patients with SMAD4-defficient PDAC treated with Metformin had significantly longer survival time than those without Metformin treatment (log-rank *P* = 0.022), with HR of death for Metformin treatment being 0.31 (95% CI = 0.14–0.68). In contrast, no significant effect of Metformin treatment on survival time was observed in patients with SMAD4-normal PDAC (Fig. 6D). These results suggest that Metformin treatment may inhibit PDAC progression and thus improve survival in patients with SMAD4-deficient PDAC.

## DISCUSSION

**Figure 7:**
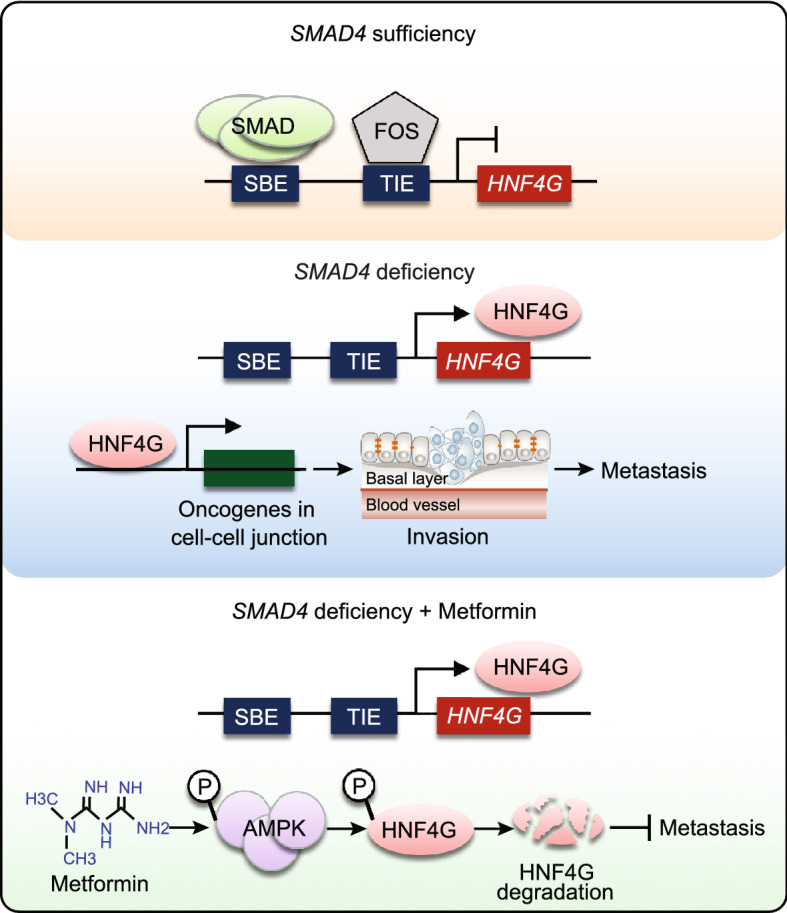
**A proposed working model for aberrant SMAD4-HNF4G in PDAC invasiveness and the effect of Metformin.** In PDAC cells where SMAD4 is sufficient, the expression of the downstream oncogene *HNF4G* that promotes PDAC invasiveness is physiologically inhibited by the SMAD complex. In PDAC cells where SMAD4 is deficient, the expression of *HNF4G* is over-activated, which evokes cancer cell invasion and metastasis. Metformin may act as a target drug repressing PDAC invasion and metastasis by activating AMPK that induces phosphorylation-ubiquitination coupled HNF4G degradation

In the present study, we have demonstrated that GWAS identified PDAC-associated *HNF4G* functions as an oncogene that plays a pivotal role in PDAC invasiveness and metastasis. Overexpression of HNF4G activates the cell-cell junction pathway, which is likely the underlying molecular mechanism for promoting PDAC invasiveness and metastasis. We have verified for the first time that HNF4G overexpression can be caused by the deficiency of SMAD4, a tumor suppressor gene that is frequently deleted or mutated in PDAC. Furthermore, we have found that panacea Metformin can suppress HNF4G activity via activating AMPK that leads to phosphorylation coupled ubiquitination degradation of HNF4G protein. Treatment of mouse PDAC xenograft and clinical PDAC patients with Metformin have revealed a curative effect in SMAD4-deficient PDAC. These findings hold the potential for clinical trials to test HNF4G pathway inhibitors including Metformin in SMAD4-deficient/HNF4G-overexpressing PDAC (Fig. 7).

Previous studies have suggested that HNF4G may act in promoting cell proliferation and invasion in several types of human cancer, such as bladder cancer (Okegawa et al., 2013), lung cancer (Wang et al., 2018) and prostate cancer (Shukla et al., 2017). Our results in the present study are consistent with these findings and extend the oncogenic role of HNF4G to pancreatic cancer. Furthermore, we have achieved several important novel findings about HNF4G. First, by combined analysis of the publically available ChIP-sequencing and RNA-sequencing data, we have identified cell-cell junction pathway may be the downstream of HNF4G that may promote PDAC cell invasion and metastasis although the ChIP-sequencing data were from hepatocellular carcinoma cells and therefore need confirmation in PDAC. Second, we have demonstrated for the first time that Metformin cause HNF4G degradation via activating AMPK and consequently suppress PDAC progression and metastasis. These findings provide the rationale for clinical trials testing HNF4G pathway inhibitors such as Metformin and others in patients with HNF4G-upregulated PDAC. In addition, because HNF4G is a transcription factor with broad regulatory functions (Lau et al., 2018), our findings might also be implicated in other types of gastrointestinal cancer with aberrant HNF4G.

Another interesting result is the identification of SMAD4 as a HNF4G transcriptional inhibitor. SMAD4 also known as DPC4 (deleted in pancreatic cancer) is a well-known tumor suppressor and inactivated in more than 50% of PDAC (Iacobuzio-Donahue et al., 2009). It has been shown that SMAD4 deficiency is associated with poor prognosis of PDAC, likely due to loss of SMAD4 control of some oncogenes (Bardeesy et al., 2006). We have demonstrated for the first time that SMAD4 can bind to the SBE site located in the HNF4G promoter region, repressing HNF4G transcription. This result is consistent with previous study showing that SMAD4 binds SBE site and recruits FOS-complex to bind TIE site, thus repressing gene expression (Kerr et al., 1990). *SMAD4* deficiency due to genomic changes such as deletion or truncated mutations may abolish its ability to repress HNF4G expression. Moreover, we have detected an inverse correlation between SMAD4 and HNF4G expression in both PDAC cell lines and clinical specimens, further supporting a negative regulation of HNF4G expression by SMAD4. A previous study reported that SMAD4 upregulates HNF4 (HNF4A/HNF4G) expression and functions as a feed-forward regulatory module to promote and stabilize the enterocyte identity (Chen et al., 2019), which is inconsistent with our result. The reason for this discrepancy is not immediately evident; however, it might be due to the differences in organ- or tumor-specific transcriptional networks (Heinz et al., 2015; Assi et al., 2019).

The panacea Metformin has been used for treating type 2 diabetes mellitus and many diseases including cancer (Libby et al., 2009); however, several problems exist in its use as anticancer agent. First, the underlying mechanism for the anticancer action remains unclear, although several hypotheses have been proposed. It has been shown that Metformin may act, in an AMPK-independent manner, through decreasing blood glucose and insulin levels, reactive oxygen species (ROS) production and DNA damage (Foretz et al., 2010; Ros and Schulze, 2013; Pernicova and Korbonits, 2014). On the other hand, Metformin may also act, in an AMPK-dependent manner, through directly inhibiting ATP synthesis which in turn activates AMPK (Miller et al., 2013). Activated AMPK may activate or inactivate some important proteins by phosphorylation (Lee et al., 2012). In the present study, we have demonstrated that Metformin can inhibit HNF4G activity in an AMPK-dependent manner: Metformin activates AMPK and the latter degrades HNF4G through phosphorylation-couples ubiquitination. Importantly, we have found that upon inhibition of HNF4G, Metformin treatment significantly represses PDAC progression and metastasis *in vitro* and *in vivo* in mice. We have also found that long-term administration of Metformin significantly reduced tumor stage and improved survival in patients with SMAD4-deficient PDAC. These results with clear acting mechanism provide additional evidence for clinical trials testing Metformin in suppressing invasion and metastasis of SMAD4-deficient PDAC. Second, some preclinical trials and *in vitro* studies have used Metformin at doses many times higher than those used in clinic (Dowling et al., 2012; Wu et al., 2016), which may limit its real application. However, in the present study, we have found that 10 μmol/L of Metformin, equivalent to the plasma level of taking 1.0 g of this panacea, is able to significantly degrade HNF4G and repress PDAC cell migration and invasion, suggesting that standard clinical Metformin dose may be effective to suppress metastasis of SMAD4-deficient PDAC. Finally, our findings suggest that the effectiveness of Metformin in suppressing PDAC progression and invasiveness depends on SMAD4 status. Over the past decade, a number of preclinical studies have reported that Metformin used solely or in combination with other remedies could inhibit PDAC progression *in vitro* or *in vivo* in some animal models (Wu et al., 2016; Chen et al., 2017). However, previous clinical trials did not show any promising results for it as an anticancer agent in PDAC (Kordes et al., 2015; Chaiteerakij et al., 2016; Reni et al., 2016). We speculate that the inconsistent results between *in vitro* assays and *in vivo* in clinical trials might result from the heterogeneity of SMAD4 status in different PDAC tumors because we have demonstrated that only SMAD4-deficient subtype of PDAC is sensitive to Metformin.

It is worth noting that we found a higher proportion of SMAD4-deficient PDAC (74.1%) in our patient set compared with that (30%–50%) reported in literature (Iacobuzio-Donahue et al., 2009). The most likely reason for this disparity might be attributable to the antibody used in the present study. This antibody targets the C-terminal of SMAD4 and therefore can detect its deficiency caused by all silencing genetic and epigenetic events rather than the deleterious mutations. Another potential reason is patient selection bias, because PDAC patients recruited in the present study all suffered from diabetes. On the other hand, one previous report has shown that the percentage of overall inactivation or loss of SMAD4 in pancreatic cancer is 60%–90% (Moustakas and Heldin, 2005) and our result (74.1%) is within the range. However, despite some disparity, the high proportion of SMAD4-deficient PDAC in our patient set may not impact our conclusion that patients with SMAD4-deficient PDAC benefit from Metformin treatment.

In summary, by using genome-wide association analysis and functional characterization, we have identified *HNF4G* as an oncogene in PDAC, whose overexpression may be caused by *SMAD4* deficiency. Overexpression of HNF4G evokes PDAC progression and invasiveness, but can be suppressed by the panacea Metformin. These findings shed light on an important mechanism underlying the acting effect of SMAD4 deficiency in PDAC development and progression and suggest that the SMAD4-HNF4G pathway may be the target for precision treatment of PDAC.

## MATERIALS AND METHODS

### Patients, biospecimens and tissue microarrays

We recruited patients from Peking Union Medical College Hospital (PUMCH, *n* = 103) and Chinese Academy of Medical Sciences Cancer Hospital (CAMSCH, *n* = 36) (Table S8). All patients were diagnosed as type 2 diabetes mellitus and PDAC. Patient survival time was measured from the date of diagnosis to the date of last follow-up or death. Whether and when a subject had died was obtained from inpatient and outpatient records, subject’s family, or through follow-up telephone calls. Informed consents were solicited from all individuals and this study was approved by PUMCH and CAMSCH. Tissue microarrays were purchased from Shanghai Outdo Biotech (HPan-Ade170Sur-01-M-138) or made using surgically removed PDAC samples at PUMCH.

### Cell lines and cell culture

PDAC cell lines BxPC-3, CFPAC-1, PANC-1, Capan-2 and MIAPaCa-2 were obtained from the China Center for Type Culture Collection while AsPC-1 and T3M4 were kind gifts from Dr. G. Yang (PUMCH). BxPC-3, AsPC-1 and Capan-2 cells were maintained in RPMI 1640, PANC-1, T3M4 and MIAPaCa-2 Cells were maintained in DMEM and CFPAC-1 cells were maintained in IMDM. All the medium were supplemented with 10% fetal bovine serum (FBS, Hyclone). These cell lines were authenticated by RNA-seq analysis of SNPs compared to exome data from Cancer Cell Line Encyclopedia. Cell lines were mycoplasma free as determined by PCR.

### Genome-wide association analysis and candidate gene selection

We combined previously published 8 PDAC GWAS (Amundadottir et al., 2009; Low et al., 2010; Petersen et al., 2010; Wu et al., 2011; Li et al., 2012; Wolpin et al., 2014; Childs et al., 2015; Klein et al., 2018) and identified 79 PDAC-associated SNPs. We then performed fine-mapping of a 2-mega base (Mb) region centered on each of these SNPs and reanalyzed our previous PDAC GWAS data (Wu et al., 2011) and found 85 SNPs in 47 genes significantly associated with PDAC (*P* < 1.0 × 10^−4^; Table S2). After excluding genes that encode anti-sense RNAs, microRNAs and genes that have been reported in our previous studies (Zheng et al., 2016; Huang et al., 2018), we selected 36 genes for further RNA interfering screen (Table S3).

### High content screening assays

The small interfering RNA (siRNA) library provided by GenePharma (Shanghai GenePharma Co., Ltd, Shanghai, China) comprised 3 individual non-overlapping siRNA designs for each gene and the repression efficiency was guaranteed by the provider. The high content screening assays were performed as previously described (Laufer et al., 2013; Laufer et al., 2014). Baseline and main effects were computed from non-targeting controls and single-gene knockdowns for each siRNA design. siRNA targeting KRAS, a known driver gene in PDAC, was used as a positive control for the screening assays.

### Immunohistochemical analysis

The sections were incubated with antibody against HNF4G (#25801-1-AP, Proteintech), SMAD4 (#46535, Cell Signaling), or p-AMPKα (#2535, Cell Signaling) at 4 °C overnight and then detected with the ABC Kit (Pierce). The labeling score of intensity was estimated as negative (0), weak (1), moderate (2) and strong (3). The extent of staining, defined as the percentage of positive stained cells, was scored as 1 (≤10%), 2 (11%–50%), 3 (51%–80%) and 4 (>80%). The total IHC score was obtained by multiplying the score of intensity and that of extent, ranking from 0 to 12.

### RNA extraction and quantitative real-time PCR analysis

RNA extraction and reverse transcription were performed using Trizol reagent (Invitrogen) and PrimeScriptTM RT reagent kit (TaKaRa), respectively. Quantitative real-time PCR (qRT-PCR) was performed in triplicate using SYBR green (Life Technologies). The primer sequences used for qRT-PCR are shown in Table S9.

### Analysis of invasion and migration

Invasion assays were performed in Millicell chambers which coated with 30 μg of Matrigel (BD Biosciences). Cells (5 × 10^4^) were added to the coated filters in serum-free medium and incubated 16–24 h; migrated cells were then fixed with methanol and stained. The migration assay was conducted in a similar fashion without coating the filters with Matrigel.

### Western blot assays

Protein extracts from cells were prepared using detergent-containing lysis buffer. Total protein (20 µg) was subjected to SDS-PAGE and transferred to PVDF membrane (Millipore). Antibody against SMAD4 (#46535), AMPK (#5831), p-AMPK (Thr172, #2535), or ubiquitin (#3933) was from Cell Signaling. Antibody against HNF4G (#25801-1-AP) or β-ACTIN (#TA-09) was from Proteintech and ZSGB-BIO. Membranes were incubated overnight at 4 °C with primary antibody and visualized with a Phototope Horseradish Peroxidase Western Blot Detection kit (Thermo Fisher). HNF4G phosphorylation assay was performed using Phos-tag Acrylamide (#F4002, APExBIO) according to the instructions.

### Chromatin immunoprecipitation coupled quantitative PCR analysis

PDAC cells were treated with formaldehyde for cross linking, followed by chromatin Immunoprecipitation (ChIP) with antibody against SMAD4 (1:100; Cell Signaling, #38454) or mouse IgG. The level of *HNF4G* DNA in the immunoprecipitation was determined by qPCR using SYBR Green and the primers for HNF4G 1st SBE was showed in Table S9.

### Construction of reporter plasmids and luciferase reporter gene assays

A reporter vector in the pGL4.10 backbone was generated containing the *HNF4G* promoter region using the restriction enzymes *Xho*I and *Hind*III. The authenticity of all the constructs was verified by sequencing. All primers used in plasmid construction are shown in Table S9. Luciferase reporter assays was performed according to the manufacturer’s instructions (Promega, Cat# E1960).

### RNA interference of gene expression

Small interfering RNA (siRNA; Table S9) targeting *HNF4G*, *SMAD4* or *PRKAA1* was purchased from GenePharma. Transfections of each siRNA (75 nmol/L) were performed with Lipofectamine 2000 (Invitrogen).

### Establishment of HNF4G-knockout cell lines by CRISPR editing

The CRISPR/Cas9 system was used to generate genomic deletion of *HNF4G* in PDAC cells. Single guide RNA (sgRNA) sequence (Table S9) targeting the genomic sequence of *HNF4G* designed using CRISPR design tool were cloned into plasmid pUC19-U6-sgRNA. The pCAG-Cas9-EGFP and pUC19-U6-sgRNA plasmids were co-transfected into PDAC cells and the fluorescent cells were sorted by flow cytometry into a 96-well plate for culture.

### Lentiviral production and infection

Lentiviral for HNF4G stably overexpression (#41208-1), SMAD4 stably overexpression (#LV-27250-1) and RNAi targeted SMAD4 (#67236-1, #67236-2) were purchased from GENECHEM as viral particles. Lentiviral for luciferase stably overexpression (pHB-LV056) was purchased from Hanbio as viral particles. Virus infection of various cell lines was performed according to the manufacturer’s instructions.

### Transient overexpression and site-directed mutagenesis

HNF4G (#23850-1) and SMAD4 (#27250-1) transient overexpressing plasmids were purchased from GENECHEM. Plasmid transfections were done using Lipofectamine 2000 (Invitrogen). Plasmids with mutant SBE or phosphorylation motifs in *HNF4G* were generated using the Muta-direct Kit (SBS) or gene synthesis by TSINGKE. The primers sequence and detail information are showed in Table S9.

### Animal experiments

Pancreatic PDAC implantation was performed on female BALB/C nude mice aged 4–6 weeks as described previously (Qiu and Su, 2013; Aiello et al., 2016). One week after PDAC transplantation, mice were randomly divided into two groups and treated with Metformin in drinking water (0.93 mg/mL) or vehicle. The body weight of animals was measured every other day. The animal experiments and procedures were approved by PUMC Animal Care and Use Committees.

### Analysis of relevant data from the public databases

Peaks annotation of ChIP-seq data (GEO accession: GSM803404) was performed as described (Yu et al., 2015). Integrative analysis was performed using ChIP-seq data and gene expression data from TCGA (Table S5). Gene Ontology analysis was performed by METASCAPE (Zhou et al., 2019) (http://metascape.org). *HNF4G* expression data in normal and PDAC tumor tissues were derived from the Gene Expression Profiling Interactive Analysis (Tang et al., 2017) (GEPIA, http://gepia.cancer-pku.cn).

### Statistical analysis

The associations between SNPs and risk of PDAC were analyzed by an additive model in a logistic regression framework with age and sex as covariates. Odds ratios (ORs) and their 95% confidence intervals (CIs) were calculated in logistic regression models adjusting for age and sex. Pearson’s and Spearman’s correlations were used to measure the correlations between groups. The correlation was considered significant when *P* < 0.05 and |r| > 0.30. Kaplan-Meier analysis and log-rank test were used to evaluate associations with survival time. Hazard ratios (HRs) and 95% CIs were calculated using Cox proportional hazards models with age, sex, tumor stage as covariates. Chi-square and Fisher’s exact tests were used to examine the difference of IHC score between groups. Student’s *t*-test were used to evaluate the difference between two groups. *P* < 0.05 was considered significant for all statistical analyses. All statistical analyses were performed using Prism 6.0 (Graphpad Software Inc.).

## Electronic supplementary material

Below is the link to the electronic supplementary material.

Supplementary material 1 (PDF 29805 kb)
