# Supplementary material for: Metformin inhibits pancreatic cancer metastasis caused by SMAD4 deficiency and consequent HNF4G upregulation
Source: Protein Cell. 2020 Jul 31;12(2):128–44. doi: 10.1007/s13238-020-00760-4 (PMC7862466; doi:10.1007/s13238-020-00760-4)
Supplement: Supplementary file 1 — Supplementary material 1 (PDF 29805 kb) [file 13238_2020_760_MOESM1_ESM.pdf]

Supplementary Figure Legends

Supplementary Figure 1. Quality control of high content screening and cell invasiveness verification.
(**A** and **B**) Pearson's correlation of the duplicated high content screening data in MIAPaCa-2 (A) and PANC-1 (B) cells. (**C**) Representative images of transwell assays showing significant differences in invasion and migration of PANC-1 cells with siRNA silencing of 9 genes identified from high content screening.

Supplementary Figure 2. SMAD4 deficiency causes upregulation of HNF4G in PDAC.
(**A**) HNF4G mRNA levels as function of HNF4G DNA copy number or mutation. Both HNF4G mutation and amplification are null or very rare in patients reported in Queensland Centre for Medical Genomics (QCMG), International Cancer Genome Consortium (ICGC), University of Texas Southwestern Medical Center (UTSW) and the TCGA project. (**B**) No correlation between HNF4G gene methylation status and the HNF4G mRNA levels. (**C**) HNF4G mRNA levels are significantly higher in PDAC with SMAD4 truncated mutations than other mutations and wild type (WT). **, $P < 0.01$ of Wilcoxon rank-sum test. Data are derived from the TCGA PDAC dataset. (**D**) The DNA sequences of SBE elements in homo sapiens and mus musculus species derived from JASPAR database. (**E** and **F**) Relative expression of reporter gene bearing the HNF4G promoter region in BxPC-3 cells with or without SMAD4 overexpression (E) and in PANC-1 cells with or without SMAD4 knockdown (F). (**G**) The plasmid constructions showing the location of SBE mutations in HNF4G reporter (upper panel) and the locations of TSS, SBEs and TIE in HNF4G promoter region (lower panel). TSS, transcriptional start site. (**H**) Enrichment of HNF4G DNA with SMAD4 antibody or IgG relative to an input control in cells with or without SMAD4 knockdown. Data are mean ± SEM from 3 independent experiments. *, $P < 0.05$ and **, $P < 0.01$ of Student t-test compared with IgG control or shControl.

Supplementary Figure 3. HNF4G regulates cell-cell junction pathway to promote PDAC metastasis.
(**A**) Immunoblot analysis of HNF4G protein levels in PDAC cells with HNF4G knockout (left) or overexpression (right). (**B**) HNF4G expression knockout significantly repressed PDAC cell migration and invasion. Left panels show representative images of transwell assays and right panels show quantitative statistic. Data represent mean ± SEM from 3 independent experiments and each had duplicate. ****, $P < 0.0001$ of Student t-test. (**C**) The mRNA expression levels of the HNF4G downstream genes in PDAC cells with HNF4G knockout. Data are mean ± SEM from 3 independent qRT-PCR determinations and each had triplicate. *, $P < 0.05$; **, $P < 0.01$; ***, $P < 0.001$ and ****, $P < 0.0001$ of Student t-test.

Supplementary Figure 4. Metformin promotes HNF4G degradation in PDAC cells.
(**A**) No effect of PIK3R1 agonist isoprenaline on phosphorylation of HNF4G and AMPKα in T3M4 cells. (**B** and **C**) Metformin promoted HNF4G degradation

but not inhibited its synthesis in CFPAC-1 cells. Metformin treatment substantially decreased the HNF4G levels with time in cells exposed to protein synthesis inhibitor cycloheximide (CHX; 20 μg/ml) compared with cells exposed to vehicle (B); Metformin treatment no longer substantially decreased HNF4G level in cells exposed to proteasome inhibitor MG132 (5 μmol/L) (C). (**D**) Metformin promotes HNF4G ubiquitination. CFPAC-1 cells were treated with Metformin (10 μmol/L) for 24 h. Cell lysates were immunoprecipitated (IP) with either control IgG or antibody against HNF4G and analyzed by immunoblotting with a ubiquitin (Ub)-specific antibody. Bottom, input from cell lysates. (**E** and **F**) Effect of Metformin on HNF4G and AMPKα phosphorylation status in HNF4G-knockout T3M4 cells with or without ectopic expression of T143R-mutated (E) or S161A (F) mutated HNF4G. (**G**) Consensus phosphorylating motif structure for AMPKα and sequence alignments of the HNF4G S382 region in human and other species.

Supplementary Figure 5. **Suppression of HNF4G-induced PDAC metastasis by Metformin is dependent on SMAD4 status.**
(**A**) Metformin significantly repressed migration and invasion in BxPC-3 and T3M4 cells in a dose-dependent manner, but not in PANC-1 cells. (**B**) Effects of the SMAD4 status on Metformin-induced migration and invasion of PDAC cells. Left panels show transwell assay results and right panels show quantitative statistic. Data represent mean ± SEM from 3 independent experiments and each had triplicates. ***, $P < 0.001$ and ****, $P < 0.0001$ and NS, not significant of Student t-test. See also Figure 5. (**C**) Mice drinking water containing Metformin (0.93 mg/ml) had similar bodyweight gain to that drinking water only during the time period of experiment.

Wang et al. Figure S1

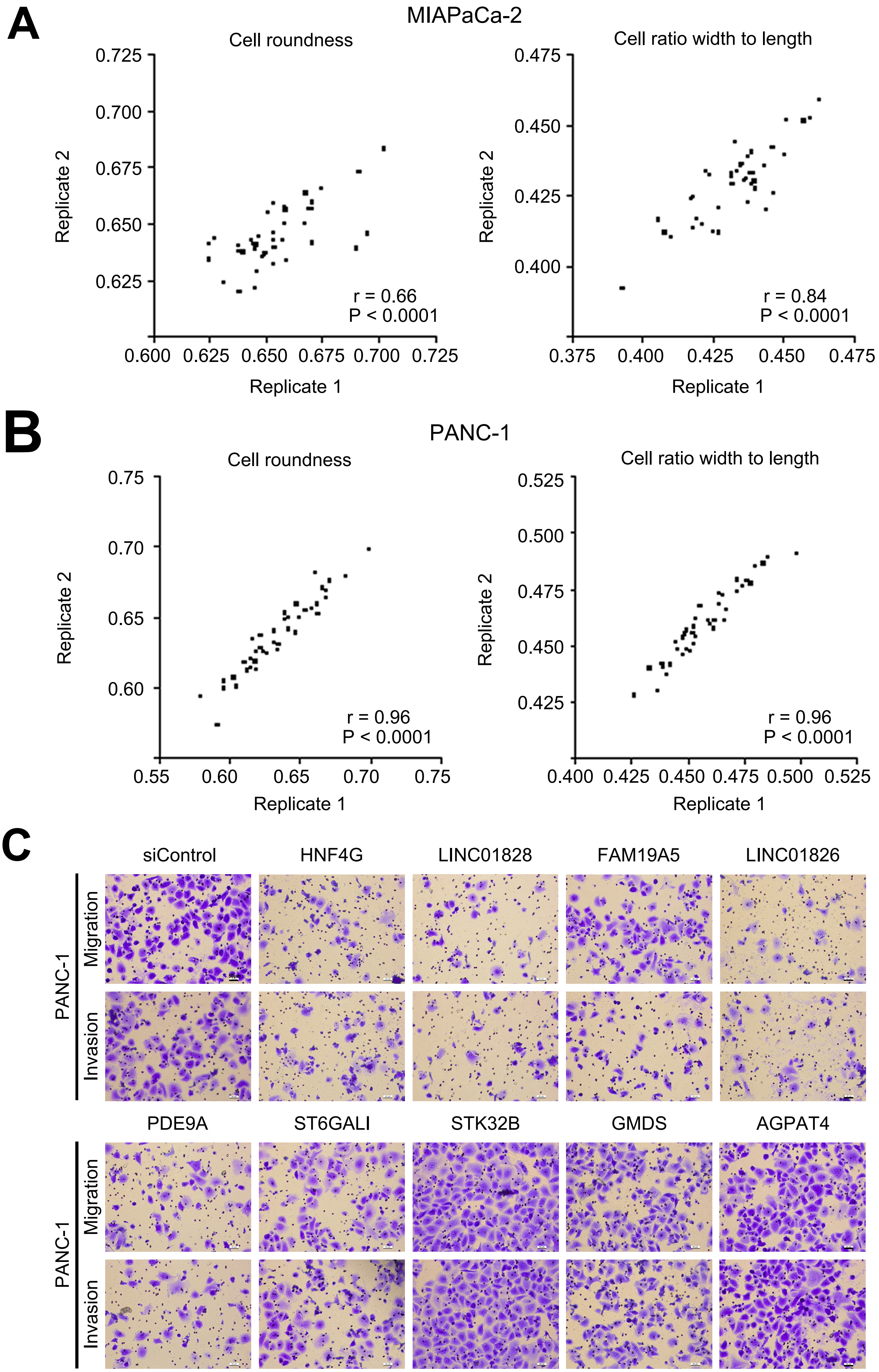

Wang et al. Figure S2

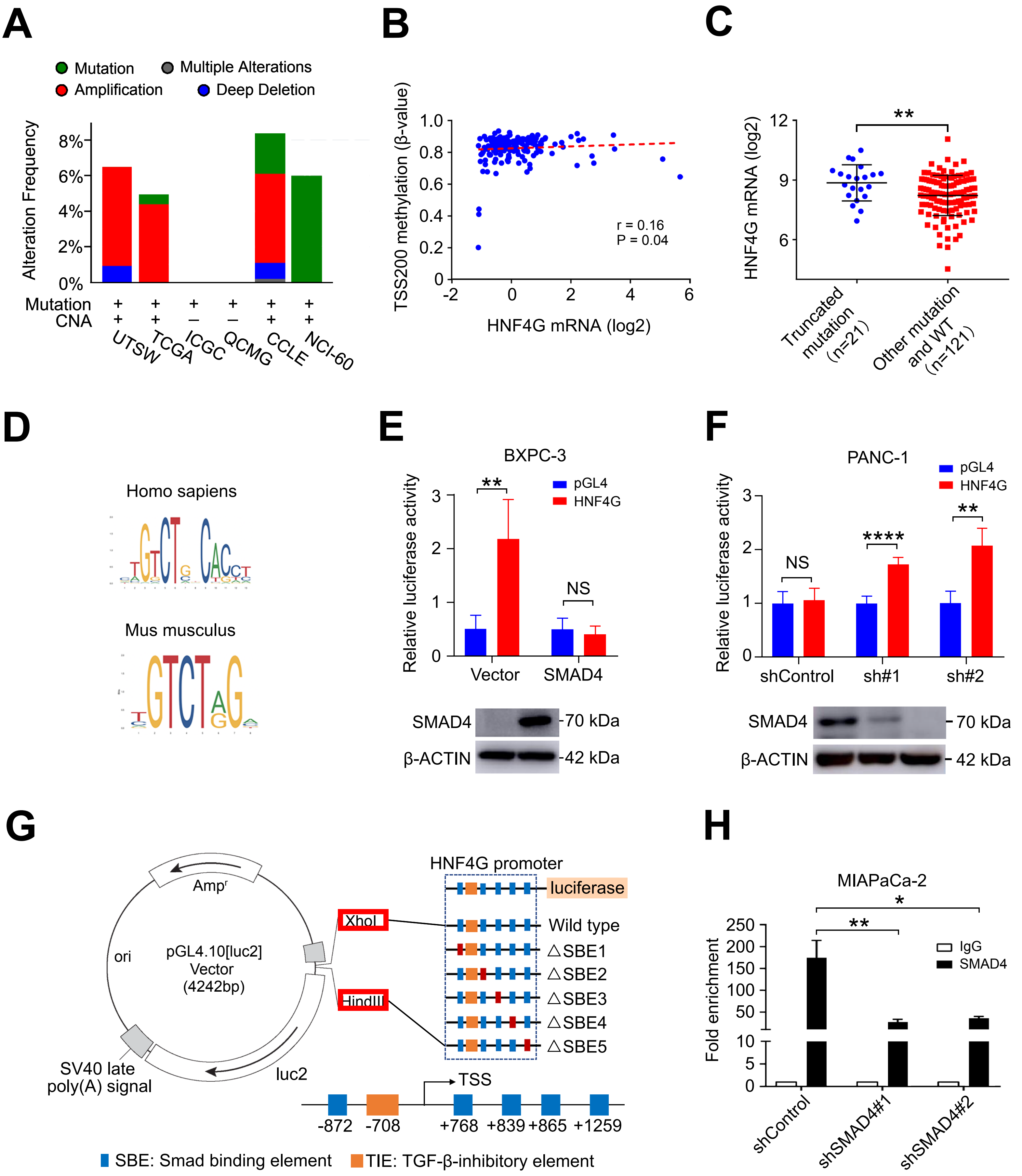

Wang et al. Figure S3

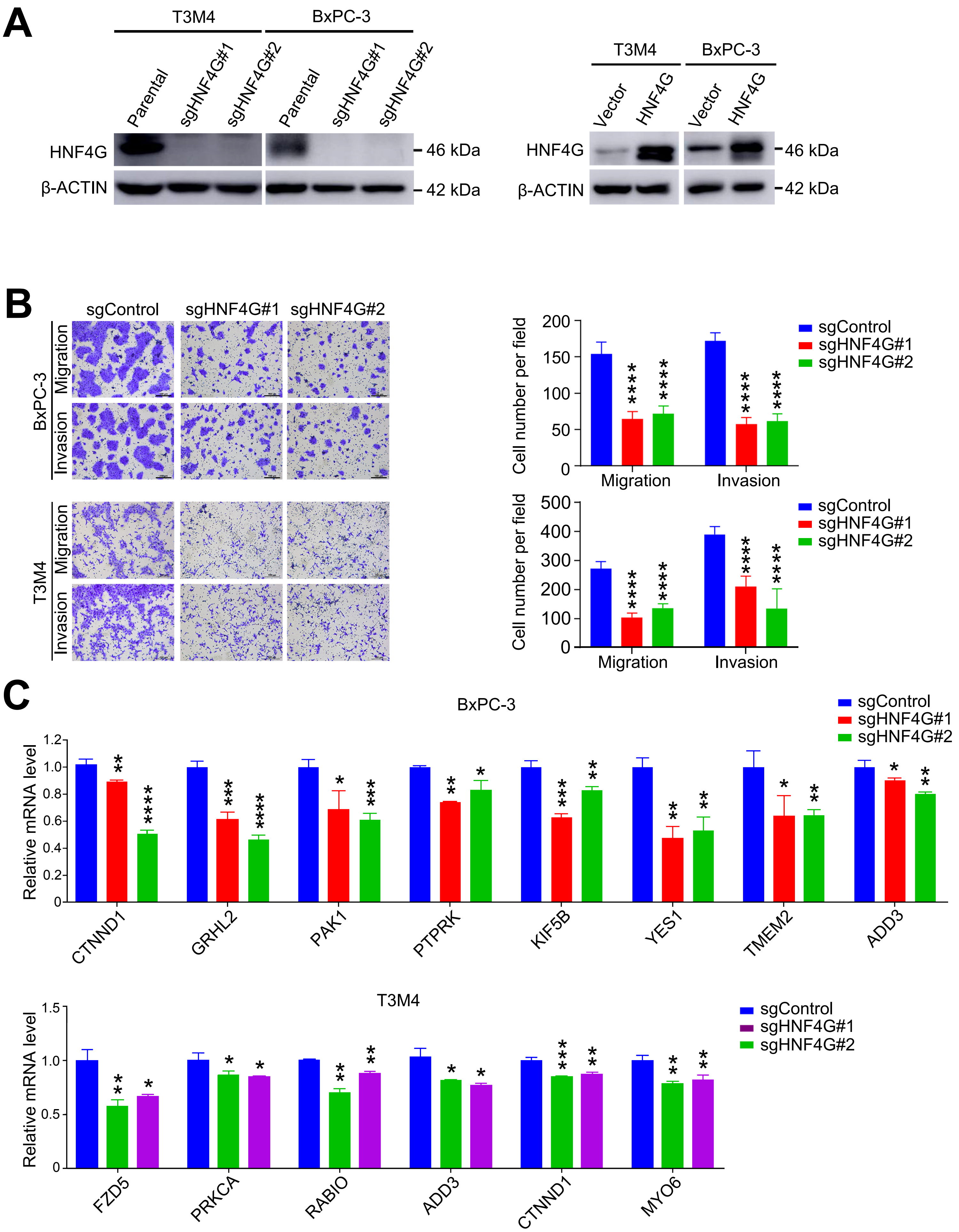

Wang et al. Figure S4

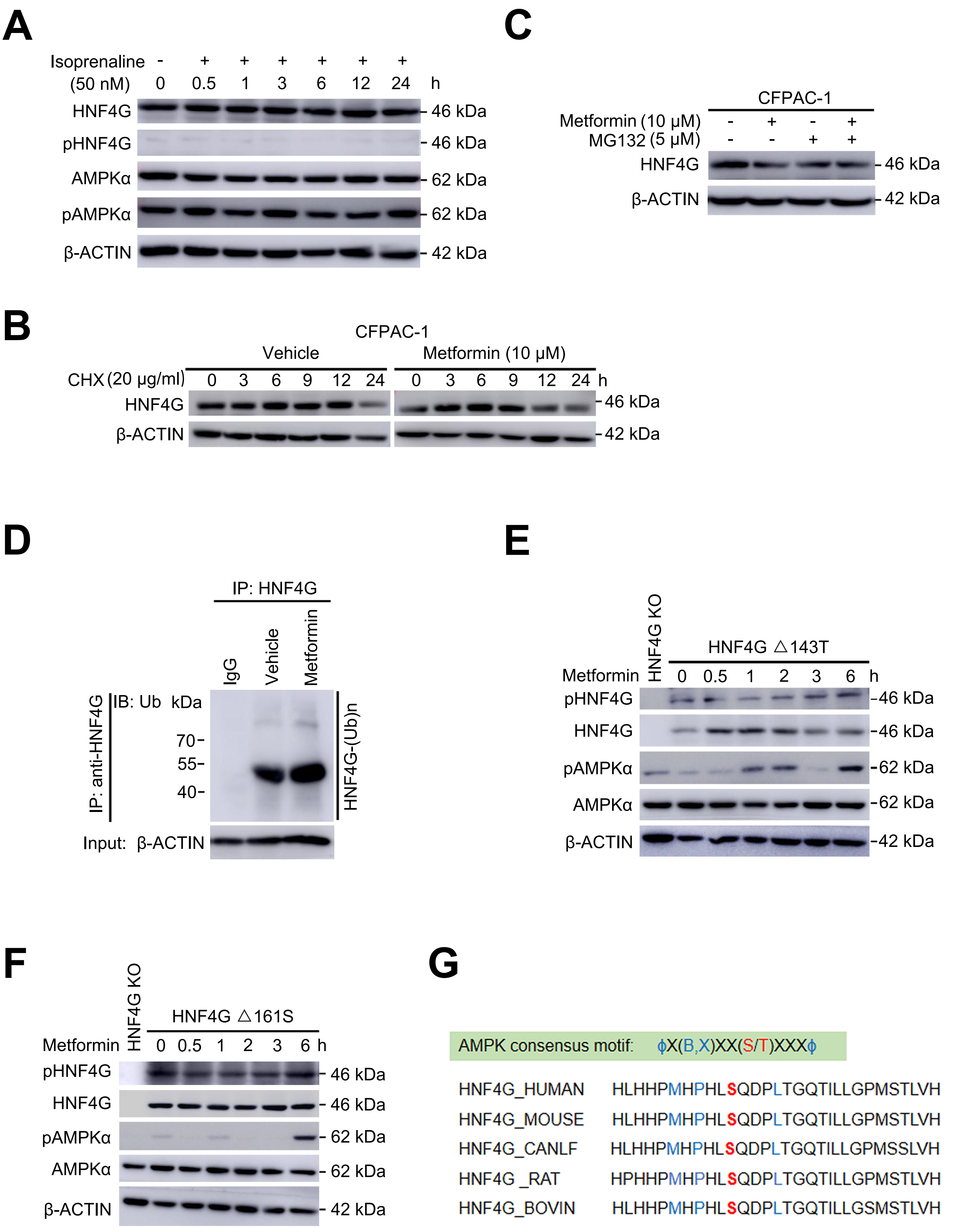

Wang et al. Figure S5

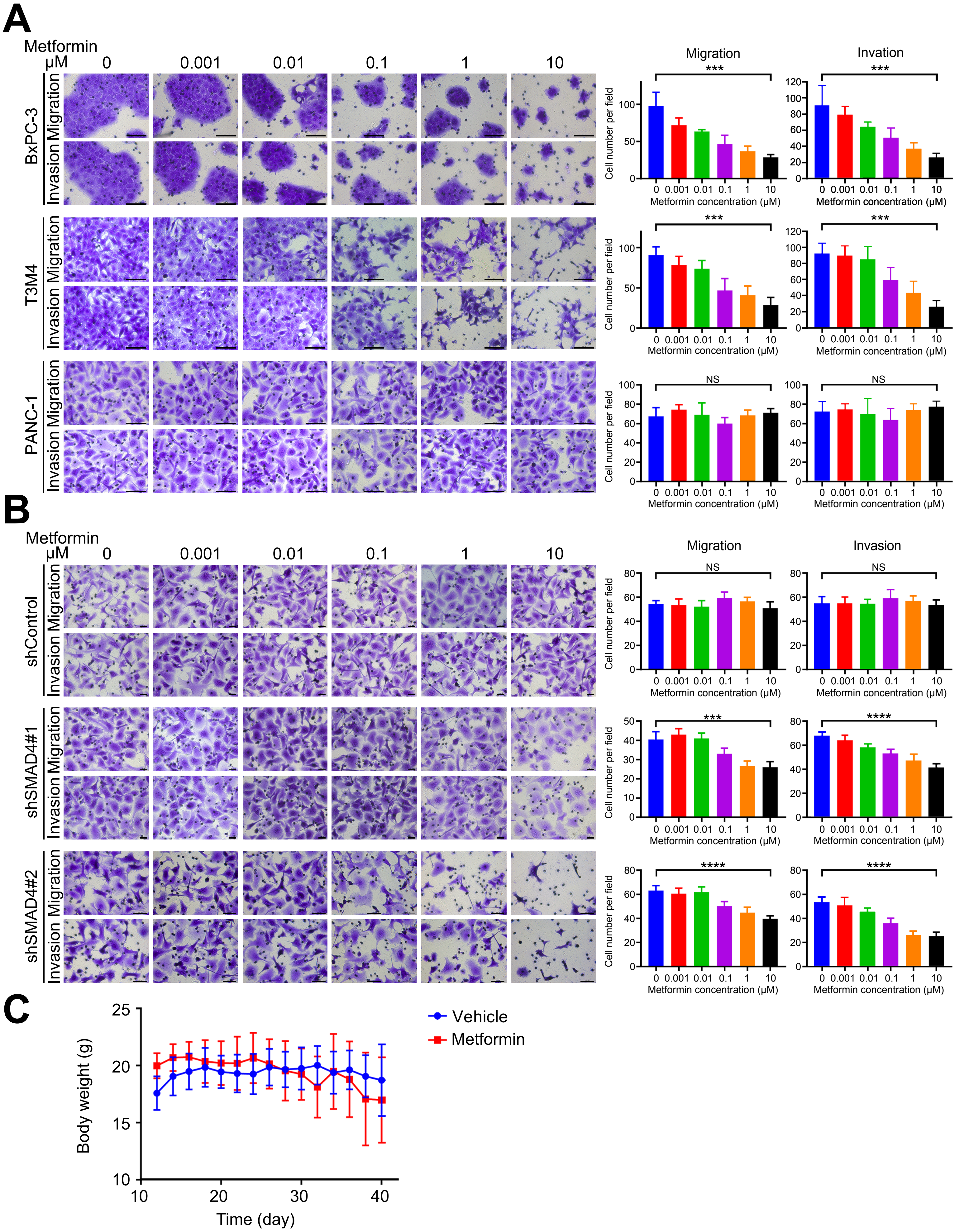

Table S1. GWAS-reported SNPs associated with susceptibility to pancreatic cancer

Year	Journal	Marker (allele)	Gene	*P* value
2009	*Nat Genet*	rs505922 (T, C)	*ABO*	5.37E-08
		rs495828 (G, T)	*ABO*	2.30E-05
		rs657152 (G, T)	*ABO*	8.28E-07
		rs630014 (C, T)	*ABO*	1.58E-07
		rs167020 (G, A)	*SHH*	2.07E-05
		rs172310 (C, A)	*SHH*	3.66E-05
		rs288746 (T, C)	*SHH*	1.45E-03
		rs8028529 (T, C)	*NA1*	8.53E-04
		rs4130461 (G, T)	*NA1*	1.32E-02
		rs4459505 (G, A)	*NA1*	3.51E-03
2010	*Nat Genet*	rs9543325 (T, C)	*NA2*	3.27E-11
		rs9564966 (G, A)	*NA2*	5.86E-08
		rs3790844 (T, C)	*NR5A2*	2.45E-10
		rs10919791 (G, A)	*NR5A2*	6.37E-10
		rs3790843 (G, A)	*NR5A2*	6.69E-09
		rs12029406 (C, T)	*NR5A2*	1.04E-07
		rs4465241 (C, T)	*NR5A2*	4.79E-07
		rs401681 (C, T)	*CLPTM1L*	3.66E-07
2010	*PLoS One*	rs9502893 (C, T)	*FOXQ1*	3.30E-07
		rs708224 (A, G)	*BICD1*	3.30E-07
		rs6464375 (C, T)	*DPP6*	4.41E-07
		rs6973850 (C, T)	*DPP6*	6.27E-07
		rs7779540 (A, G)	*DPP6*	4.58E-07
		rs11242679 (A, G)	*FOXQ1*	2.40E-06
		rs7750826 (A, G)	*FOXQ1*	2.57E-06
		rs10487687 (A, G)	*DPP6*	3.35E-06
		rs11242674 (A, G)	*FOXQ1*	3.46E-06
		rs6711606 (G, T)	*RNF149*	4.02E-06
		rs10088262 (A, G)	*FAM91A1*	4.30E-06
		rs7832232 (A, G)	*RNF5P1*	5.10E-06
		rs6736997 (A, C)	*ARL4C*	5.85E-06
		rs225190 (A, G)	*MYO1D*	5.99E-06
		rs4663158 (C, T)	*ARL4C*	6.91E-06
		rs2039553 (A, G)	*NDFIP2*	7.01E-06
		rs1427593 (A, G)	*THSD7B*	7.10E-06
		rs3016539 (A, G)	*PARK2*	7.28E-06
		rs12615966 (C, T)	*LOC284998*	7.44E-06
		rs2257205 (A, G)	*RNF43*	7.74E-06
		rs6879627 (C, T)	*LOC731559*	8.12E-06
		rs4924935 (C, T)	*PRPSAP2*	8.15E-06
		rs1737947 (C, T)	*PRPSAP2*	8.49E-06
		rs1886449 (C, T)	*LOC730242*	9.24E-06

		rs1585440 (A, C)	*LOC387933*	9.28E-06
		rs4683235 (C, T)	*LTF*	9.93E-06
2012	*Carcinogenesis*	rs7310409 (G, A)	*HNF1A*	1.00E-05
		rs2464196 (C, T)	*HNF1A*	6.30E-05
		rs1169300 (G, A)	*HNF1A*	7.90E-05
		rs735396 (A, G)	*HNF1A*	1.00E-04
		rs7953249 (A, G)	*HNF1A*	2.30E-04
		rs1805100 (G, A)	*HNF4G*	3.20E-05
		rs2977926 (T, G)	*HNF4G*	3.40E-04
		rs4794758 (C, T)	*HNF1B*	7.30E-04
		rs2043136 (T, C)	*TGFBR2*	1.60E-05
		rs3773650 (C, A)	*TGFBR2*	7.90E-05
		rs1062225 (A, G)	*MAPK8*	6.30E-04
		rs13396983 (G, A)	*BCL2L11*	6.80E-04
		rs2015454 (C, T)	*BCL2L11*	7.00E-04
2011	*Nat Genet*	rs372883 (A, G)	*BACH1*	2.24E-13
		rs2255280 (A, C)	*DAB2*	4.18E-10
		rs12413624 (A, T)	*PRLHR*	5.12E-11
		rs1547374 (A, G)	*TFF1*	3.71E-13
		rs4885093 (A, G)	*NA2*	1.57E-12
		rs9573163 (C, G)	*NA2*	5.14E-13
		rs5768709 (A, G)	*FAM19A5*	1.41E-10
2014	*Nat Genet*	rs2736098 (T, C)	*TERT*	9.78E-14
		rs6971499 (C, T)	*LINC-PINT*	2.98E-12
		rs7190458 (A, G)	*BCAR1*	1.13E-10
		rs9581943 (A, G)	*PDX1*	2.35E-09
		rs16986825 (T, C)	*ZNRF3*	1.18E-08
		rs1561927 (C, T)	*MIR1208, PVT1*	1.30E-07
2015	*Nat Genet*	rs11655237 (T, C)	*LINC00673*	1.42E-14
		rs7214041 (T, C)	*LINC00673*	2.88E-14
		rs1486134 (G, T)	*ETAA1*	3.36E-09
		rs17688601 (A, C)	*SUGCT*	1.41E-08
		rs9854771 (A, G)	*TP63*	2.35E-08
2018	*Nat Commun*	rs13303010 (G, A)	*NOC2L*	8.36E-14
		rs2941471 (G, A)	*HNF4G*	6.60E-10
		rs4795218 (A, G)	*HNF1B*	1.32E-08
		rs1517037 (T, C)	*GRP*	3.28E-08

Table S2. The information on 85 significant SNPs

Chr	SNP	Location	Annotation	OR	*P* value
1	rs3790854	200000125	NR5A2	1.28	3.01E-05
1	rs3790843	200010824	NR5A2	1.26	7.31E-05
2	rs2035565	67619656	ETAA1	1.33	5.39E-07
2	rs1486133	67642021	ETAA1	1.30	2.77E-06
2	rs3770657	67630980	ETAA1	1.30	3.09E-06
2	rs6726820	67181166	LINC01799	0.64	3.22E-06
2	rs1601529	67594837	(-)	1.29	5.84E-06
2	rs6740919	67620055	ETAA1	1.29	6.60E-06
2	rs12713595	67523685	BC040863, LINC01826	1.28	8.04E-06
2	rs7592026	67595139	(-)	1.28	8.65E-06
2	rs13415002	67611823	(-)	1.27	1.30E-05
2	rs12477464	67595239	(-)	0.78	1.31E-05
2	rs1032157	67601816	(-)	1.28	1.33E-05
2	rs961328	67555446	(-)	0.78	1.33E-05
2	rs6710832	67601942	(-)	1.27	1.52E-05
2	rs18122	67654434	(-)	1.27	2.17E-05
2	rs661435	30189304	(-)	1.26	2.80E-05
2	rs1483351	67520575	BC040863, LINC01826	1.26	4.45E-05
2	rs13016812	67624302	ETAA1	1.25	6.13E-05
3	rs1991902	30189304	(-)	0.78	2.67E-05
3	rs56328838	190026785	CLDN1	1.56	8.08E-05
5	rs2255280	39394989	DAB2	0.77	7.61E-06
5	rs2686172	39395533	DAB2	0.77	9.86E-06
5	rs2855509	39396944	DAB2	0.78	2.35E-05
5	rs2367584	39412568	DAB2	0.78	3.09E-05
6	rs7774091	162353966	PARK2, PRKN	1.30	5.78E-06
6	rs117582557	161610212	AGPAT4	0.34	5.01E-05
6	rs139838744	161635358	AGPAT4	0.35	5.96E-05
6	rs78627167	1721845	GMDS, AK024936	1.38	9.66E-05
8	rs17299691	76103211	(-)	2.17	3.64E-12
8	rs7822330	129416116	BC009730	1.50	1.93E-06
8	rs830772	76352578	HNF4G	0.76	6.59E-06
8	rs7012884	38919407	ADAM9	1.64	2.00E-05
8	rs7835278	75765947	PI15, AK024242	0.39	4.66E-05
9	rs2520006	136622110	VAV2, AK123314	1.48	8.26E-05
10	rs12413624	120278944	(-)	1.31	1.53E-06
10	rs1316856	120267557	(-)	1.31	2.17E-06
10	rs1002526	120304184	(-)	1.30	2.86E-06
10	rs11198490	120339418	(-)	1.29	1.55E-05
10	rs11198509	120370663	(-)	1.26	5.23E-05
13	rs140362176	67072770	PCDH9	0.14	8.71E-23
13	rs75865077	67073577	PCDH9	0.14	1.22E-22

13	rs117015133	67073653	PCDH9	0.14	1.22E-22
13	rs75432155	67073075	PCDH9	0.14	1.44E-22
13	rs140786904	67072306	PCDH9	0.14	1.94E-22
13	rs139235823	67074209	PCDH9	0.14	1.96E-22
13	rs117841163	67074434	PCDH9	0.14	4.54E-22
13	rs117959615	67075365	PCDH9	0.14	1.55E-21
13	rs118177383	67076895	PCDH9	0.13	4.26E-21
13	rs150948452	67072153	PCDH9	0.18	5.04E-21
13	rs75523662	67077040	PCDH9	0.12	5.26E-21
13	rs78167153	67077578	PCDH9	0.12	2.68E-20
13	rs139429747	67071311	PCDH9	0.46	8.45E-11
13	rs147547981	67071300	PCDH9	0.59	6.76E-07
13	rs4885093	73910026	(-)	1.31	1.68E-06
13	rs9573163	73908846	(-)	1.30	3.18E-06
13	rs287553	73904230	(-)	1.30	3.71E-06
13	rs9318163	73897389	(-)	1.29	7.11E-06
13	rs2265326	66061553	(-)	0.73	3.22E-05
13	rs17090071	73929428	(-)	1.27	7.97E-05
15	rs4924065	36703076	(-)	6.34	8.21E-38
15	rs1846137	36155088	DPH6-AS1	0.74	2.97E-05
16	rs17205188	54685198	(-)	0.69	7.03E-05
17	rs1304660	70226409	(-)	5.95	3.34E-27
17	rs4793412	70226159	(-)	0.39	5.65E-06
17	rs9893376	18823578	PRPSAP2	0.58	9.35E-06
17	rs7225065	57373202	(-)	0.25	3.69E-05
17	rs11655237	70400166	Mir_584, LIN00673	1.30	4.71E-05
20	rs6104137	44014286	SYS1, TP53TG5	0.63	8.64E-14
21	rs372883	30717737	BACH1	0.73	8.80E-08
21	rs1153294	30701096	BACH1	0.74	1.17E-07
21	rs2027605	30726773	BACH1	0.74	1.37E-07
21	rs2832290	30728863	BACH1	0.74	1.52E-07
21	rs1153287	30685611	BACH1	0.74	1.82E-07
21	rs117214	30720834	BACH1	0.74	1.98E-07
21	rs1153280	30678072	BACH1	0.74	3.28E-07
21	rs1547374	43778895	TFF2, TFF1	0.75	1.43E-06
21	rs1236481	30697673	BACH1	0.75	1.77E-06
21	rs363445	30986229	GRIK1, GRIK1-AS2	0.76	1.01E-05
21	rs16982189	29728865	(-)	1.37	1.68E-05
21	rs2839486	43768197	TFF2	0.76	2.58E-05
21	rs4920087	43776839	TFF2, TFF1	0.76	3.44E-05
21	rs12626719	30902661	GRIK1	0.70	4.82E-05
21	rs2269130	44078191	PDE9A, BC033260	0.78	6.39E-05
22	rs5768709	48929569	FAM19A5, LOC284933	1.34	3.93E-06

Chr, chromosome; OR, odds ratio; (-), gene desert region

Table S3. High content screening results

	Cell Roundness		Cell Ratio	
Gene	MIAPaCa-2	Panc-1	MIAPaCa-2	Panc-1
HNF4G	1.17	1.13	1.27	1.13
LINC01826	1.10	1.14	1.13	1.12
STK32B	1.14	1.07	1.22	1.11
GMDS	1.08	1.13	1.09	1.10
FAM19A5	1.06	1.14	1.09	1.13
PDE9A	1.08	1.11	1.11	1.10
ST6GALI	1.09	1.10	1.11	1.12
LINC01828	1.09	1.07	1.12	1.12
AGPAT4	1.08	1.09	1.15	1.06
CLDN1	1.04	1.11	1.03	1.09
DAB2	1.05	1.09	1.04	1.08
SYS1-DBNPD2	1.03	1.09	1.05	1.11
PLI5	1.03	1.09	1.06	1.07
GRIK1	1.08	1.04	1.11	1.08
ADAM9	1.06	1.06	1.09	1.06
SYS1	1.04	1.08	1.03	1.11
VAV2	1.01	1.10	1.01	1.09
GRIK1-AS2	1.04	1.06	1.07	1.06
GLIS3	1.04	1.05	1.04	1.03
HSD17B12	1.03	1.06	1.03	1.03
LINC00824	1.03	1.04	1.01	1.04
LRPPRC	1.04	1.03	1.06	1.06
TPM1	1.02	1.04	1.06	1.04
DPH6-AS1	1.04	1.02	1.02	1.05
ZNF385D	1.03	1.03	1.07	1.04
PCDH9	1.02	1.03	1.06	0.99
TFF1	1.01	1.04	1.01	1.03
LINC017992	1.03	1.02	1.04	1.04
RCL1	1.00	1.05	1.00	1.02
NR5A2	1.04	1.00	1.07	1.01
KRAS	1.00	1.04	1.06	1.01
TFF2	1.01	1.01	1.00	1.02
PRPK2	1.00	1.01	1.06	1.03
TP53-TG5	1.02	0.98	1.03	0.99
ETAA1	1.00	0.99	1.02	0.99
PRPSAP	0.98	1.01	0.97	1.02
L3MBTL1	0.96	1.02	0.94	1.04

Table S4. QIAGENE-predicted 115 transcriptional factors that might bind to HFN4G promoter or enhancer

ZIC2	*CREM*	*ZNF207*	*TFE3*
BCL6	*THAP11*	*SP3*	*L3MBTL2*
SOX13	*KLF11*	*ZHX2*	*RNF2*
MLX	*GATAD2B*	*MXD4*	*NFIA*
ZEB2	*TGIF2*	*PPARG*	*KLF6*
NR3C1	*RAD51*	*SSRP1*	*ZNF511*
HNF4A	*KLF16*	*ZNF610*	*NRF1*
CTCF	*CEBPA*	*GLIS1*	*NR2F1*
DRAP1	*IRF1*	*MXD3*	*ZFP37*
ZNF792	*ZBTB26*	*RCOR2*	*ZGPAT*
RING1	*PRDM1*	*KDM1A*	*HDAC2*
ZNF580	*MGA*	*ZZZ3*	*TCF7*
REST	*TFAP4*	*SUZ12*	*KLF9*
RBBP5	*MXI1*	*HBP1*	*ZFX*
MIER3	*SAP130*	*EED*	*ZNF423*
GLIS2	*TAF1*	*ZBTB20*	*GABPB1*
DMAP1	*MAX*	*SOX5*	*MBD1*
EP300	*EBF1*	*BHLHE40*	*FOXP2*
HIC1	*E2F1*	*FOXA3*	*SMAD4*
MLLT1	*GATAD1*	*MNT*	*TRIM28*
HNRNPUL1	*NCOR1*	*HMG20A*	*ELF3*
FOXA2	*POLR2A*	*ZNF394*	*TBP*
ARID4B	*PATZ1*	*ZBTB8A*	*TBL1XR1*
ZNF2	*EZH2*	*ZNF501*	*DNMT3B*
ZNF48	*GATAD2A*	*DPF2*	*RAD21*
YY1	*PHF8*	*HNF1A*	*RARA*
SLC30A9	*ZNF687*	*ZNF692*	*KAT2B*
IKZF2	*ZNF366*		

Table S5. 293 downstream target genes of HNF4G

Gene	Chr.	Start	End	Annotation
CORO2A	chr9	100945154	100945871	intron (NM_052820, intron 1 of 11)
CREB3L3	chr19	4153154	4153779	promoter-TSS (NM_032607)
SLC22A23	chr6	3378241	3378702	intron (NM_001286456, intron 3 of 3)
OCLN	chr5	68813641	68814019	intron (NM_001205255, intron 2 of 6)
RNF128	chrX	105962034	105962324	intron (NM_024539, intron 1 of 6)
C1orf115	chr1	220871902	220872361	3' UTR (NM_024709, exon 2 of 2)
PCK2	chr14	24562951	24563802	promoter-TSS (NM_004563)
GOLGA4	chr3	37284924	37285422	intron (NM_001172713, intron 1 of 22)
GRHL2	chr8	102585508	102585867	intron (NM_024915, intron 5 of 15)
CHN2	chr7	29376553	29377213	intron (NM_004067, intron 1 of 12)
C1orf106	chr1	200865604	200866368	intron (NM_001142569, intron 1 of 9)
SPTBN1	chr2	54799411	54799820	intron (NM_178313, intron 1 of 30)
SUCLG2	chr3	67551210	67551432	intron (NM_003848, intron 7 of 10)
LOC201651	chr3	151487907	151488434	promoter-TSS (NR_026915)
NQO1	chr16	69760078	69761071	promoter-TSS (NM_001025433)
LOC100129034	chr9	127113238	127113616	3' UTR (NM_001166167, exon 10 of 10)
C9orf41	chr9	77616741	77617424	intron (NM_152420, intron 3 of 7)
ROCK2	chr2	11485385	11485862	promoter-TSS (NM_004850)
CCDC68	chr18	52626408	52626943	promoter-TSS (NM_025214)
EFNA2	chr19	1291637	1292154	intron (NM_001405, intron 1 of 3)
ATE1	chr10	123602926	123603316	intron (NM_001001976, intron 8 of 11)
NCOA2	chr8	71244768	71245258	intron (NM_006540, intron 1 of 22)
FRMD3	chr9	86087063	86087577	intron (NM_001244959, intron 1 of 14)
CDKN2AIP	chr4	184365001	184366012	promoter-TSS (NM_017632)
GSR	chr8	30580288	30580587	intron (NM_001195103, intron 1 of 11)
TJP2	chr9	71793355	71793742	intron (NM_001170630, intron 1 of 18)
CDH1	chr16	68824048	68824509	intron (NM_004360, intron 2 of 15)
CALML4	chr15	68484430	68484668	non-coding (NR_104583, exon 2 of 2)
ARHGAP12	chr10	32218177	32218567	promoter-TSS (NM_018287)
PCGF5	chr10	92979528	92980204	promoter-TSS (NM_032373)
TMEM45B	chr11	129687475	129687954	intron (NM_138788, intron 1 of 5)
GALNT7	chr4	173647211	173647636	intron (NM_001034845, intron 5 of 12)
FNIP2	chr4	159733728	159734100	intron (NM_020840, intron 1 of 16)
MAGI3	chr1	113936805	113937256	intron (NM_152900, intron 1 of 20)
ALDOB	chr9	104195370	104195985	intron (NM_000035, intron 1 of 8)
TMEM2	chr9	74373732	74374098	intron (NM_013390, intron 1 of 23)
ELOVL7	chr5	60097462	60097836	intron (NM_024930, intron 1 of 8)
NHSL1	chr6	138825083	138825870	intron (NM_020464, intron 1 of 6)
PTPRK	chr6	128602140	128602504	intron (NM_001135648, intron 3 of 30)
NHEJ1	chr2	219976216	219976737	intron (NM_024782, intron 5 of 7)
APOL6	chr22	36044305	36044792	5' UTR (NM_030641, exon 1 of 3)
ASAH2	chr10	52153499	52153907	intron (NM_147156, intron 6 of 10)

SH3RF2	chr5	145361478	145361806	intron (NM_152550, intron 2 of 9)
PPFIBP2	chr11	7546634	7547050	intron (NM_003621, intron 1 of 23)
TCF7L2	chr10	114729258	114729668	intron (NM_001146274, intron 4 of 13)
RBM47	chr4	40543295	40543581	intron (NM_001098634, intron 2 of 6)
APOB	chr2	21265630	21266406	intron (NM_000384, intron 2 of 28)
MYO6	chr6	76601385	76601621	intron (NM_004999, intron 27 of 34)
EFTUD1	chr15	82524222	82524569	intron (NM_001040610, intron 5 of 17)
GNA11	chr19	3093850	3094189	promoter-TSS (NM_002067)
FMO5	chr1	146683141	146683475	intron (NM_001461, intron 5 of 8)
RFFL	chr17	33402337	33402703	intron (NR_037713, intron 1 of 6)
SLC37A1	chr21	43946409	43946937	intron (NM_018964, intron 4 of 20)
TREH	chr11	118550202	118550565	promoter-TSS (NM_007180)
ACSL5	chr10	114154070	114154677	intron (NM_016234, intron 1 of 20)
CYP3A4	chr7	99370164	99370565	intron (NM_001202855, intron 3 of 12)
CAMK2D	chr4	114557506	114557920	intron (NM_172115, intron 3 of 16)
FZD5	chr2	208712454	208713054	intron (NM_001080475, intron 7 of 7)
HNF4A	chr20	42999153	42999656	intron (NM_001030003, intron 1 of 9)
C4orf19	chr4	37432957	37433328	intron (NM_001144990, intron 4 of 6)
HSD17B2	chr16	82093455	82094205	intron (NM_002153, intron 1 of 4)
ANXA13	chr8	124733017	124733281	intron (NM_004306, intron 1 of 10)
GLCE	chr15	69514223	69514594	intron (NM_015554, intron 2 of 4)
ABCG2	chr4	89028468	89029335	intron (NM_001257386, intron 9 of 15)
ZDHHC20	chr13	22027866	22028182	intron (NR_104487, intron 1 of 12)
TRIM10	chr6	30122468	30122884	intron (NM_006778, intron 6 of 6).4
ATP7B	chr13	52553090	52553470	intron (NM_001005918, intron 1 of 16)
ARHGEF12	chr11	120206581	120207040	promoter-TSS (NM_001198665)
EPS8L3	chr1	110306393	110306974	promoter-TSS (NM_024526)
FAM83G	chr17	18884905	18885325	intron (NM_001039999, intron 3 of 5)
GIPC2	chr1	78511476	78511954	5' UTR (NM_017655, exon 1 of 6)
LPIN2	chr18	2984759	2985330	intron (NM_014646, intron 1 of 19)
C1orf210	chr1	43751397	43751732	promoter-TSS (NM_001164829)
PRKCA	chr17	64455871	64456407	intron (NM_002737, intron 2 of 16)
MFHAS1	chr8	8746240	8746503	intron (NM_004225, intron 1 of 2)
ALDH18A1	chr10	97394821	97395606	intron (NM_001017423, intron 5 of 17)
HNRNPF	chr10	43894713	43895096	intron (NM_001098204, intron 1 of 3)
ACSS2	chr20	33462537	33462851	promoter-TSS (NM_001242393)
TMPRSS2	chr21	42847994	42848236	intron (NM_001135099, intron 8 of 13)
KALRN	chr3	124330328	124330698	intron (NM_007064, intron 1 of 26)
KIF5B	chr10	32312213	32312513	intron (NM_004521, intron 15 of 25)
GPR160	chr3	169755458	169755960	promoter-TSS (NM_014373)
AQP11	chr11	77304834	77305314	intron (NM_173039, intron 1 of 2)
FBLN7	chr2	112927105	112927573	intron (NM_153214, intron 3 of 7)
ANG	chr14	21155367	21156495	intron (NM_002937, intron 1 of 1)
LIMA1	chr12	50582045	50582399	intron (NM_016357, intron 9 of 10)

ATP8B1	chr18	55465867	55466252	intron (NM_005603, intron 1 of 27)
ERBB3	chr12	56473706	56474028	promoter-TSS (NM_001982)
PKP2	chr12	33044930	33045176	intron (NM_001005242, intron 1 of 12)
SLC30A10	chr1	220100626	220100945	intron (NR_046437, intron 1 of 3)
ATP2C2	chr16	84448551	84448885	intron (NM_001286527, intron 6 of 27)
ABCC6P1	chr16	18588043	18588329	intron (NR_003569, intron 4 of 9)
SLC25A13	chr7	95950367	95950910	intron (NR_027662, intron 1 of 16)
ZNF512B	chr20	62600054	62600781	intron (NM_020713, intron 1 of 16)
PDSS1	chr10	26989865	26990149	intron (NM_014317, intron 1 of 11)
THOC2	chrX	122866805	122867048	promoter-TSS (NM_001081550)
NNMT	chr11	114080677	114081011	intron (NM_001018011, intron 4 of 6)
SEMA4G	chr10	102723519	102724033	3' UTR (NM_018121, exon 20 of 20)
TMEM82	chr1	16068718	16069109	promoter-TSS (NM_001013641)
SULT1B1	chr4	70620887	70621296	intron (NM_014465, intron 1 of 7)
HTRA2	chr2	74756144	74757346	exon (NM_181575, exon 1 of 12)
LGALS4	chr19	39309725	39310260	intron (NM_001398, intron 3 of 9)
GOLM1	chr9	88708650	88709044	intron (NM_177937, intron 1 of 9)
ARAP1	chr11	72477213	72477916	intron (NM_006645, intron 2 of 6)
ARHGAP21	chr10	25011972	25012338	intron (NM_020824, intron 1 of 25)
SLC7A9	chr19	33360439	33360835	promoter-TSS (NM_001126335)
CGN	chr1	151492821	151493199	intron (NM_020770, intron 4 of 20)
PLCH1	chr3	155340513	155340723	intron (NM_014996, intron 1 of 21)
TMEM144	chr4	159132541	159132849	5' UTR (NM_018342, exon 2 of 13)
UROD	chr1	45477621	45478007	promoter-TSS (NM_000374)
RILPL1	chr12	123983062	123983446	exon (NM_178314, exon 4 of 7)
CEBPG	chr19	33890820	33891316	intron (NM_001166056, intron 10 of 12)
SLC44A1	chr9	108053606	108054070	intron (NM_080546, intron 1 of 15)
USP8	chr15	50715589	50716042	promoter-TSS (NM_001128610)
TBC1D25	chrX	48407281	48407575	intron (NM_002536, intron 3 of 5)
KBTBD12	chr3	127694398	127694780	intron (NM_207335, intron 4 of 4)
FBXO44	chr1	11708555	11708882	3' UTR (NM_012168, exon 6 of 6)
NR1I2	chr3	119544560	119545113	3' UTR (NM_002093, exon 12 of 12)
ANK3	chr10	62182476	62183033	intron (NM_001204403, intron 2 of 43)
DDAH1	chr1	85904036	85904445	intron (NM_001134445, intron 2 of 6)
NBEAL1	chr2	203905953	203906338	intron (NM_001114132, intron 2 of 54)
MAP3K13	chr3	185046386	185046929	intron (NM_001242317, intron 2 of 12)
RNF103	chr2	86859838	86860176	intron (NM_001198954, intron 2 of 7)
TSPAN3	chr15	77359650	77359997	intron (NM_198902, intron 1 of 5)
TBC1D8B	chrX	106045736	106046047	promoter-TSS (NM_198881)
STK39	chr2	169047057	169047775	intron (NM_013233, intron 1 of 17)
MYO5B	chr18	47721021	47721605	5' UTR (NM_001080467, exon 1 of 40)
AGPAT9	chr4	84527113	84527370	TTS (NM_032717)
SLC35A3	chr1	100435051	100435663	promoter-TSS (NM_001271685)
ST3GAL3	chr1	44223108	44223342	intron (NM_001270463, intron 2 of 7)

KLF5	chr13	73559756	73560126	intron (NM_006346, intron 16 of 17)
PAK1	chr11	77170396	77170693	intron (NM_001128620, intron 1 of 15)
PLEKHA6	chr1	204317393	204317652	intron (NM_014935, intron 1 of 22)
GOLPH3L	chr1	150640261	150640711	intron (NM_018178, intron 2 of 4)
ANKS4B	chr16	21271987	21272738	intron (NM_001888, intron 9 of 9)
RBM27	chr5	145593083	145593368	intron (NM_018989, intron 1 of 20)
C9orf129	chr9	96075250	96075590	intron (NM_001282394, intron 28 of 29)
ADD3	chr10	111767278	111767625	promoter-TSS (NM_001121)
SLC35D2	chr9	99153502	99154147	intron (NM_153695, intron 4 of 4)
ENTPD5	chr14	74462633	74463069	intron (NM_001249, intron 3 of 15)
DOPEY2	chr21	37574513	37574974	intron (NM_005128, intron 4 of 36)
FGD4	chr1	216038405	216038720	intron (NM_206933, intron 44 of 71)
LTBP3	chr16	67595892	67596318	promoter-TSS (NM_006565)
SH3RF1	chr1	48688174	48688585	promoter-TSS (NM_001135181)
LCOR	chr9	99252888	99254245	TTS (NM_014282)
CDHR5	chr4	74464761	74465316	intron (NM_201431, intron 2 of 10)
HMGCS2	chr17	41052371	41052950	promoter-TSS (NM_000151)
FCHO2	chr4	100495675	100496262	5' UTR (NM_000253, exon 2 of 19)
CNOT6	chr7	130985929	130986231	intron (NM_001145354, intron 2 of 18)
ZDHHC9	chr6	47488017	47488268	intron (NM_012120, intron 2 of 17)
DPP8	chr4	25156438	25156928	exon (NM_016955, exon 5 of 11)
AVL9	chr10	52645211	52645593	promoter-TSS (NM_014576)
ARL5B	chr13	41575786	41576099	intron (NM_172373, intron 1 of 8)
PLD1	chr7	45075896	45076298	intron (NM_001029835, intron 1 of 9)
LRRC8B	chr17	46029488	46029981	3' UTR (NM_024320, exon 2 of 2)
NAT1	chr5	133984084	133984471	promoter-TSS (NM_001252231)
MECOM	chr21	18899515	18899775	intron (NM_001207063, intron 1 of 4)
SNX4	chr16	15073740	15074046	intron (NM_001285449, intron 1 of 16)
BARD1	chr11	57532013	57532381	intron (NM_001085467, intron 1 of 16)
DENND1B	chr14	92364860	92365109	intron (NM_006329, intron 4 of 10)
DRAP1	chr2	231904621	231905144	intron (NM_001144994, intron 1 of 2)
IYD	chrX	106361798	106362154	promoter-TSS (NM_001171080)
DNAJC22	chr1	15911047	15911877	exon (NM_024758, exon 1 of 7)
PLA2G12B	chr1	26324313	26325007	promoter-TSS (NM_000437)
KIAA1161	chr18	774877	775204	intron (NM_005433, intron 1 of 11)
LYPD6	chr4	57107603	57107963	intron (NM_020722, intron 2 of 10)
KCNE3	chr4	48018102	48018495	promoter-TSS (NM_207330)
HKDC1	chr9	139971858	139972098	promoter-TSS (NM_207309)
PRDM10	chr2	219283499	219284178	promoter-TSS (NM_007127)
ACOX1	chr12	32686995	32687343	intron (NM_139241, intron 1 of 16)
SMEK2	chr11	65318963	65319423	intron (NM_021070, intron 8 of 26)
GK3P	chr4	170338869	170339154	intron (NM_001199400, intron 28 of 32)
CDC14B	chr10	98623639	98624155	intron (NM_001170765, intron 2 of 7)
RNF166	chr11	624760	625214	promoter-TSS (NM_001171968)

MFSD9	chr1	120308924	120309414	intron (NM_005518, intron 1 of 9)
VKORC1	chr4	111119679	111120061	promoter-TSS (NM_001130721)
SLC35D1	chr2	88427347	88428313	promoter-TSS (NM_001443)
ACER3	chr17	55162863	55164094	intron (NM_001242903, intron 1 of 11)
STRN	chr6	131958171	131958568	promoter-TSS (NM_005021)
TCEA2	chr14	73571032	73571270	intron (NM_021239, intron 10 of 18)
PTPRH	chr1	207131048	207131303	TTS (NM_001122979)
OAT	chr1	184761531	184761926	3' UTR (NM_052966, exon 14 of 14)
SLC26A3	chr4	88296824	88297328	intron (NM_016245, intron 2 of 6)
TAOK3	chr18	61078047	61078304	intron (NM_004869, intron 2 of 10)
ICK	chr2	26267202	26267552	intron (NM_016131, intron 1 of 5)
MRPL35	chr2	191331516	191331812	intron (NM_017694, intron 3 of 7)
PARP4	chr15	94903625	94903845	intron (NM_001159644, intron 2 of 9)
AIM1	chr2	165794144	165794645	intron (NM_173512, intron 4 of 9)
WAPAL	chr13	113365744	113366076	intron (NM_015205, intron 1 of 29)
CLDN12	chr10	98397396	98398036	intron (NM_152309, intron 8 of 16)
ENTPD7	chr19	5839440	5839987	promoter-TSS (NM_000150)
TMEM181	chr7	197478	197847	intron (NM_020223, intron 2 of 9)
HEATR5A	chr6	116382492	116382872	promoter-TSS (NM_002031)
BCL2L14	chr15	52346621	52346928	intron (NM_002748, intron 3 of 5)
MYO1A	chr9	74736490	74736779	intron (NM_001242507, intron 1 of 13)
SLC13A2	chr16	18812602	18813380	promoter-TSS (NM_015161)
CPD	chr12	100673653	100673908	intron (NM_017988, intron 1 of 17)
MPP5	chr12	100949499	100950021	intron (NM_001206992, intron 7 of 8)
CES2	chr6	54794293	54794791	intron (NM_001010872, intron 4 of 4)
SMAGP	chr1	67635679	67635932	intron (NM_144701, intron 3 of 10)
LONP2	chr5	142186974	142187539	intron (NM_015071, intron 1 of 22)
PRKAA1	chr4	170589521	170589842	intron (NM_001243374, intron 1 of 11)
EIF3A	chr10	101939349	101939834	intron (NM_001100626, intron 3 of 11)
TNFSF15	chr7	141804658	141804946	intron (NM_004668, intron 47 of 47)
AKR1B10	chr2	38904875	38905246	intron (NM_138801, intron 2 of 6)
CYP4F2	chr9	80619441	80619753	intron (NM_002072, intron 1 of 6)
TNPO3	chr2	234620078	234620400	intron (NM_001072, intron 1 of 4)
STXBP3	chr2	112842644	112843114	intron (NM_032824, intron 8 of 18)
ADH4	chr2	85581214	85581943	promoter-TSS (NM_001135022)
SYTL5	chr10	99275363	99275663	intron (NM_024954, intron 1 of 2)
CMPK1	chr7	99317899	99318279	intron (NM_000765, intron 3 of 12)
C11orf54	chr6	82957435	82957807	promoter-TSS (NM_015525)
BAZ2B	chr9	115984775	115985126	intron (NM_001859, intron 1 of 4)
LONRF3	chr11	60552612	60552823	promoter-TSS (NM_206893)
ELOVL6	chr5	175972706	175973075	intron (NM_001171976, intron 1 of 31)
FABP1	chr3	124710847	124711217	intron (NM_020733, intron 12 of 16)
AKAP1	chr4	69961928	69962364	promoter-TSS (NM_001074)
ENPP3	chr2	44065558	44066502	promoter-TSS (NM_022436)

PSEN1	chr6	33377611	33378284	promoter-TSS (NM_002636).2
PIGR	chrX	95976425	95976679	intron (NM_007309, intron 1 of 26)
EDEM3	chr3	111395610	111395922	intron (NM_153268, intron 1 of 3)
HSD17B11	chr11	2368702	2368969	intron (NR_108080, intron 1 of 2)
VPS4B	chr10	72164992	72165525	intron (NM_004096, intron 1 of 2)
RAB10	chr18	72208896	72209670	intron (NM_032649, intron 1 of 11)
MFSD6	chr6	46760821	46761191	promoter-TSS (NM_005588)
MCTP2	chr16	75561730	75562102	TTS (NM_024533)
SLC38A11	chr5	72251563	72252030	promoter-TSS (NM_138782)
ATP11A	chr5	179921067	179921480	promoter-TSS (NM_015455)
TM9SF3	chrX	128960103	128960424	intron (NM_001008222, intron 3 of 9)
FUT6	chr15	65809862	65810186	promoter-TSS (NM_197960)
FAM20C	chr7	32555623	32555936	intron (NM_015060, intron 1 of 15)
FRK	chr10	18948963	18949457	intron (NM_178815, intron 1 of 5)
MAPK6	chr3	171524296	171524564	intron (NM_001130081, intron 1 of 25)
GDA	chr1	90053355	90053776	intron (NM_001134476, intron 7 of 7)
ARL6IP1	chr8	18027739	18027994	promoter-TSS (NM_001160179)
SCYL2	chr3	169286135	169286462	intron (NM_004991, intron 1 of 16)
GAS2L3	chr3	125201212	125201660	intron (NR_073435, intron 5 of 12)
FAM83B	chr2	215674399	215674750	promoter-TSS (NM_001282549)
IL23R	chr1	197589639	197590088	intron (NM_001195215, intron 10 of 22)
ARHGAP26	chr11	65686684	65687014	promoter-TSS (NM_001135635)
CLCN3	chr6	150689839	150690209	promoter-TSS (NM_001164695)
ERLIN1	chr12	49741474	49741988	intron (NM_024902, intron 1 of 2)
LOC93432	chr10	74714361	74714838	promoter-TSS (NM_032562)
GALM	chr9	34374871	34375476	intron (NM_020702, intron 1 of 1)
GNAQ	chr2	150276553	150276979	intron (NM_194317, intron 1 of 4)
UGT1A5	chr11	74176435	74176740	intron (NM_005472, intron 1 of 2)
TMEM87B	chr10	70982075	70982477	intron (NM_025130, intron 1 of 17)
RETSAT	chr11	129843664	129844068	intron (NM_020228, intron 1 of 21)
UBTD1	chr17	73974949	73975940	promoter-TSS (NM_004035)
CYP3A7	chr2	55844573	55845269	promoter-TSS (NM_020463)
IBTK	chr4	166177631	166177806	intron (NM_001161521, intron 4 of 14)
SLC31A1	chr9	99328576	99329348	intron (NM_003671, intron 1 of 12)
MS4A10	chr16	88766804	88767093	intron (NM_001171816, intron 2 of 5)
CDHR2	chr2	103340455	103340919	intron (NM_032718, intron 4 of 5)
MUC13	chr16	31104640	31105006	intron (NM_024006, intron 1 of 2)
UGT2B7	chr1	67519172	67520200	exon (NM_015139, exon 1 of 12)
ABCG5	chr11	76600473	76600788	intron (NM_018367, intron 1 of 10)
PHF1	chr2	37142739	37142999	intron (NM_003162, intron 3 of 17)
DIAPH2	chr20	62689097	62689385	intron (NM_198723, intron 1 of 10)
PLCXD2	chr19	55717923	55718291	exon (NM_001161440, exon 3 of 18)
CD81	chr10	126107254	126107723	promoter-TSS (NM_000274)
EIF4EBP2	chr7	107420034	107420602	intron (NM_000111, intron 11 of 20)

CNDP1	chr12	118810752	118811281	promoter-TSS (NM_016281)
MEP1A	chr6	52926344	52926981	promoter-TSS (NM_014920)
CHST5	chr2	86462409	86462841	intron (NM_001164732, intron 3 of 4)
KCTD3	chr13	25086156	25087110	intron (NM_006437, intron 1 of 33)
CTCF	chr6	106960147	106960701	exon (NM_001624, exon 1 of 20)
SLC5A9	chr10	88210612	88210922	intron (NM_015045, intron 15 of 18)
HABP4	chr7	90039016	90039277	intron (NM_001185073, intron 2 of 2)
RASSF6	chr10	101433119	101433546	intron (NM_020354, intron 3 of 12)
G6PC	chr6	158957032	158957450	promoter-TSS (NM_020823)
MTTP	chr14	31888941	31890506	promoter-TSS (NM_015473)
MKLN1	chr12	12235082	12235403	intron (NM_030766, intron 2 of 5)
CD2AP	chr12	57443715	57444101	promoter-TSS (NM_005379)
SEPSECS	chr17	26802211	26802615	intron (NM_001145975, intron 1 of 11)
A1CF	chr17	28732057	28732439	intron (NM_001199775, intron 2 of 20)
ELF1	chr14	67708133	67708605	intron (NM_022474, intron 1 of 14)
CCM2	chr16	66969178	66969623	exon (NM_003869, exon 1 of 12)
PRR15L	chr12	51664441	51664815	promoter-TSS (NM_001033873)
SEC24A	chr16	48277979	48278386	promoter-TSS (NM_031490)
CXADR	chr5	40784303	40784706	intron (NM_206907, intron 1 of 9)
PDXDC1	chr10	120840200	120840697	promoter-TSS (NM_003750)
CTNND1	chr9	117566789	117567103	intron (NM_005118, intron 1 of 3)
TC2N	chr7	134212029	134212326	promoter-TSS (NM_020299)
C2orf72	chr19	16008631	16009142	promoter-TSS (NM_001082)
RBM41	chr7	128694770	128695388	promoter-TSS (NR_002187)
AGMAT	chr1	109288926	109289269	promoter-TSS (NM_007269)
PAFAH2	chr4	100065361	100065690	promoter-TSS (NM_000670)
YES1	chrX	37878361	37878639	intron (NM_138780, intron 1 of 16)
KIAA1211	chr1	47800162	47800599	intron (NM_001136140, intron 1 of 4)
NIPAL1	chr11	93474976	93475380	promoter-TSS (NM_024116)
UAP1L1	chr2	160471836	160472328	intron (NM_013450, intron 1 of 36)
VIL1	chrX	118110082	118110348	promoter-TSS (NM_001289109)

Chr, chromosome; TSS, transcriptional start site.

Table S6. HNF4G target genes enriched in the cell-cell junction pathway

Gene	Pearson r	*P* value
CDH1	0.45	2.07E-10
CDHR2	0.53	3.30E-14
CDHR5	0.59	1.83E-18
CTNND1	0.49	5.35E-12
GRHL2	0.45	3.11E-10
MPP5	0.44	5.59E-10
OCLN	0.56	5.77E-16
STRN	0.50	1.52E-12
LIMA1	0.54	7.77E-15
RAB10	0.43	1.99E-09
CGN	0.43	5.94E-09
KIF5B	0.50	9.47E-13
MYO6	0.53	3.91E-14
PDXDC1	0.64	8.52E-22
SPTBN1	0.45	2.87E-10
TJP2	0.44	6.85E-10
TMEM2	–0.35	1.57E-06
USP8	0.43	2.50E-09
CCM2	–0.43	1.49E-09
CXADR	0.58	2.09E-17
ANK3	0.43	2.71E-09
CLDN12	0.49	3.86E-12
FZD5	0.52	1.11E-13
MAGI3	0.49	5.15E-12
CD2AP	0.49	4.69E-12
PKP2	0.40	2.28E-08
SMAGP	0.44	1.02E-09
ADD3	0.53	1.93E-14
PAK1	0.49	4.61E-12
PTPRK	0.43	2.25E-09
YES1	0.44	4.93E-10
PRKCA	0.56	1.53E-08

Table S7. Scansite predicted HNF4G phosphorylation motifs and the corresponding kinases

Kinase	HNF4G motif	Score	Site	Site sequence
PDPK1	PDK1 Binding	0.94	D97	STRRSTFdGSNIPSI
CLK2	Clk2 Kinase	0.84	S43	FRRSIRKsHVYSCRF
IPCEF1	PIP3-binding PH	0.83	F35	SCDGCKGfFRRSIRK
PRKAA1	AMP Kinase	0.71	T106	SNIPSINtLAQAEVR
AURKA	Aurora A	0.69	S188	LLGATKRsMMYKDIL
PIK3R1	p85 SH3 mode1	0.68	P376	STPETPLpSPPQGSG
AKT1	Akt Kinase	0.66	T91	NERDRIStRRSTFDG
HCLS1	Cortactin SH3	0.65	P376	STPETPLpSPPQGSG
MAPK3	Erk1 Kinase	0.65	T350	HLSQDPLtGQTILLG
CDK5	Cdk5 Kinase	0.64	S121	SRQISVSsPGSSTDI
GSK3A	GSK3 Kinase	0.62	S114	LAQAEVRsRQISVSS
GSK3B	GSK3-improved Acidophilic	0.60	S114	LAQAEVRsRQISVSS
MAPK1	Erk D-domain	0.58	L288	SRGRFGElLLLLPTL
PRKACG	Protein Kinase A	0.56	S39	CKGFFRRsIRKSHVY
CAMK2G	Calmodulin dependent kinase 2	0.55	T95	RISTRRStFDGSNIP
AURKB	Aurora B	0.55	S39	CKGFFRRsIRKSHVY
ABL1	Abl SH3	0.54	P376	STPETPLpSPPQGSG
YWHAZ	41701 Mode 1	0.53	S118	EVRSRQIsVSSPGSS
PRKCZ	PKC zeta	0.52	S39	CKGFFRRsIRKSHVY
PRKDC	DNA PK	0.52	S345	HPMHPHLsQDPLTGQ
GRB2	Grb2 SH2	0.51	Y273	VQIGLEDyINDRQYD
PRKCE	PKC epsilon	0.51	S39	CKGFFRRsIRKSHVY
ITK	Itk Kinase	0.50	Y201	ILLLGNNyVIHRNSC
PRKCD	PKC delta	0.50	S43	FRRSIRKsHVYSCRF
NCK1	Nck 2nd SH3	0.48	P376	STPETPLpSPPQGSG
CDK1	CDK1 motif 1 - [ST]Px[KR]x	0.43	S121	SRQISVSsPGSSTDI
SRC	Src SH3	0.41	P376	STPETPLpSPPQGSG
PRKCA	PKC alpha/beta/gamma	0.36	S39	CKGFFRRsIRKSHVY
ATM	ATM Kinase	0.30	S345	HPMHPHLsQDPLTGQ

Table S8. Baseline characteristics and clinical data of patients with PDAC in this study

	No. of patients	SMAD4– (N = 103)*		SMAD4+ (N = 36)*	
		Metformin (%)	Others (%)	Metformin (%)	Others (%)
Sex					
Male	92	24 (26.1)	44 (47.8)	9 (9.8)	15 (16.3)
Female	47	9 (19.2)	26 (55.3)	4 (8.5)	8 (17.0)
Age (years)					
≤ 60	49	13 (26.5)	21 (42.9)	5 (10.2)	10 (20.4)
> 60	90	20 (22.2)	49 (54.5)	8 (8.9)	13 (14.4)
Type 2 Diabetes					
Recent-onset	52	16 (30.8)	23 (44.2)	6 (11.5)	7 (13.5)
Long-standing	79	17 (21.5)	39 (49.4)	7 (8.9)	16 (20.2)
Unknown	8	0 (0.0)	8 (100.0)	0 (0.0)	0 (0.0)
Differentiation					
Well	21	6 (28.6)	7 (33.3)	3 (14.3)	5 (23.8)
Moderate	80	17 (21.2)	44 (55.0)	7 (8.8)	12 (15.0)
Poor	23	8 (34.8)	11 (47.8)	1 (4.4)	3 (13.0)
Unknown	15	2 (13.3)	8 (53.4)	2 (13.3)	3 (20.0)
Stage					
I	26	9 (34.6)	11 (42.3)	1 (3.9)	5 (19.2)
II	65	15 (23.1)	32 (49.2)	8 (12.3)	10 (15.4)
III	13	1 (7.7)	8 (61.5)	2 (15.4)	2 (15.4)
IV	18	6 (33.3)	9 (50.0)	2 (11.1)	1 (5.6)
Unknown	17	2 (11.8)	10 (58.8)	0 (0.0)	5 (29.4)
Survival status					
Death	56	10 (17.9)	30 (53.5)	6 (10.7)	10 (17.9)
Survival	82	22 (26.8)	40 (48.8)	7 (8.5)	13 (15.9)
Unknown	1	1 (100.0)	0 (0.0)	0 (0.0)	0 (0.0)

*SMAD4–, immunohistochemistry (IHC) score = 0; SMAD4+, IHC score > 0.

Table S9. Sequences of primers, siRNAs and sgRNAs used in this study

Primer	Used for	Sequence (5'-3')
HNF4G-promoter-F	PCR	CCGCTCGAGGTAAACAACACACCTATGAAAGATT
HNF4G-promoter-R	PCR	CCCAAGCTTATGAGAGATGGATGGAAGCTGAGAC
HNF4G-F	Real-time PCR	GAGGTTTGGAGAGTTGCTTCTGC
HNF4G-R	Real-time PCR	GATGACTGCCATCATTGGAAGCC
GAPDH-F	Real-time PCR	GTCTCCTCTGACTTCAACAGCG
GAPDH-R	Real-time PCR	ACCACCCTGTTGCTGTAGCCAA
CDHR2-F	Real-time PCR	GGCTCCTACAACATCTTCGTCC
CDHR2-R	Real-time PCR	GGTTGAAGAGCAGACGGCTGTT
CDHR5-F	Real-time PCR	CCTTGTCCACAAGCACTATGGC
CDHR5-R	Real-time PCR	GAGGAACGCCTGGTTGTCAAAG
CTNND1-F	Real-time PCR	GAATCGGTGCTCACCAACACAG
CTNND1-R	Real-time PCR	ATCCTTCTGCCCAATCTCAGCC
GRHL2-F	Real-time PCR	CGCCTATCTCAAAGACGACCAG
GRHL2-R	Real-time PCR	CCAGGGTGTACTGAAATGTGCC
MPP5-F	Real-time PCR	TCCTCTTATCTCCAACGCACAAG
MPP5-R	Real-time PCR	CTGCTCAGCAACCTTATCGTGG
OCLN-F	Real-time PCR	ATGGCAAAGTGAATGACAAGCGG
OCLN-R	Real-time PCR	CTGTAACGAGGCTGCCTGAAGT
STRN-F	Real-time PCR	GTCCTGTTCAGCAGATGGCACT
STRN-R	Real-time PCR	CTGCTCACTAGATCCACAGAGG
LIMA1-F	Real-time PCR	AATAGCCTGGCAGTCCGTTCCA
LIMA1-R	Real-time PCR	GGAATCTGGACTTAGTGGCTTGG
RAB10-F	Real-time PCR	AAGGCGTTCCTCACGTTAGCTG
RAB10-R	Real-time PCR	GGAACAGGAGAATGCTCAGCAG
CGN-F	Real-time PCR	CAAGGAGGATCTTAGAGCCACC
CGN-R	Real-time PCR	TGGCGAGTATCTCCAGCACTAG
KIF5B-F	Real-time PCR	GAGTTAGCAGCATGTCAGCTTCG
KIF5B-R	Real-time PCR	GCATCGACAGATTCCTCCAACTG
MYO6-F	Real-time PCR	CCTGACCACTTAGCAGAGTTGG
MYO6-R	Real-time PCR	TTTAATGCAGGCTTCAGCTCGATA
PDXDC1-F	Real-time PCR	GTTCAGCCCTTTGATGACCGCA
PDXDC1-R	Real-time PCR	CTTCCTGCTTGAACTCTTCACGC
SPTBN1-F	Real-time PCR	GTCACATGCCTACCAGCAGTTC
SPTBN1-R	Real-time PCR	CTCCTTCCAAGGTGGTAGGCAT
TJP2-F	Real-time PCR	ATTAGTGCGGGAGGATGCCGTT
TJP2-R	Real-time PCR	TCTGCCACAAGCCAGGATGTCT
TMEM2-F	Real-time PCR	GGAATAGGACTGACCTTTGCCAG
TMEM2-R	Real-time PCR	TTCTGACCACCCTGAAAGCCGT
USP8-F	Real-time PCR	GATCGTACCAGGACTGCCTTCA
USP8-R	Real-time PCR	GCAGATGAAGGAGCCATTTCCG
CCM2-F	Real-time PCR	GCCTCTATCCACGAGTTCTGCA
CCM2-R	Real-time PCR	AAGTGCTGGCTGTCCTTCTCAG
CXADR-F	Real-time PCR	GCTTGCTCTAGCGCTCATTGGT
CXADR-R	Real-time PCR	GCTCTTTGGAGGTGGCACATCT
ANK3-F	Real-time PCR	AAAGGACTGCCTCAAACAGCGG
ANK3-R	Real-time PCR	CTAAGGATGCGAAGCTCTGTCG

CLDN12-F	Real-time PCR	GAGCAGTGACTGCCTGATGTAC
CLDN12-R	Real-time PCR	GCACATTCCAATCAGGCAGAGC
FZD5-F	Real-time PCR	TGGAACGCTTCCGCTATCCTGA
FZD5-R	Real-time PCR	GGTCTCGTAGTGGATGTGGTTG
MAGI3-F	Real-time PCR	GCAGTTTCCAGTAGGTGCTGATG
MAGI3-R	Real-time PCR	GTGGAAAAGGCATAGGCTGAGG
CD2AP-F	Real-time PCR	CCAAAGCCTGAACTGATAGCTGC
CD2AP-R	Real-time PCR	GGACTTGTGGAGCTGCTGGTTT
PKP2-F	Real-time PCR	CAGGTGCTGAAGCAAACCAGAG
PKP2-R	Real-time PCR	CCAAAGTGCTGGGATTACAGGC
SMAGP-F	Real-time PCR	AAGATGGAGCCAGCACAGCACT
SMAGP-R	Real-time PCR	CATAGGTGACGTAGCTGCCTTTG
ADD3-F	Real-time PCR	GGGTCACTTGAAGAACAGGAGG
ADD3-R	Real-time PCR	TCTCCAAGTGCAACCACACCATG
PAK1-F	Real-time PCR	GTGAAGGCTGTGTCTGAGACTC
PAK1-R	Real-time PCR	GGAAGTGGTTCAATCACAGACCG
PTPRK-F	Real-time PCR	CACAGCCATCAATGTCACCACC
PTPRK-R	Real-time PCR	CACCTTTGGCTTGTGCTGGTCT
YES1-F	Real-time PCR	CTCCTGAAGCTGCACTGTATGG
YES1-R	Real-time PCR	CCTGTATCCTCGCTCCACTTGT
PRKCA-F	Real-time PCR	GCCTATGGCGTCCTGTTGTATG
PRKCA-R	Real-time PCR	GAAACAGCCTCCTTGGACAAGG
ΔSBE1-F	Site-directed mutagenesis	GGCTGCAGGTCGACTCTAGAGGATCCCGCCACCAT GGACATGGCAAATTACAGTGAAGTT
ΔSBE1-R	Site-directed mutagenesis	CCATTTACCGTAAGTTATGTGCTAGCTCATTTGTCG TCATCATCCTTATAGTCCTTATCA
T143R-F	Site-directed mutagenesis	CAACATCCCCTCCATTAACAGACTGGCACAAGCTG AAGTTCGGTCTCGCCAGATCTCAGTCTCAA
T143R-R	Site-directed mutagenesis	GAACTTCAGCTTGTGCCAGTCTGTTAATGGAGGGG ATGTTGCTGCCATCAAATGTGCTTCTTCTG
S161A-F	Site-directed mutagenesis	TCTCAGTCTCAAGCCCTGGGGCAAGCACTGACATA AACGTTAAGAAAATTGCAAGTATTG
S161A-R	Site-directed mutagenesis	AACGTTTATGTCAGTGCTTGCCCCAGGGCTTGAGA CTGAGATCTGGCGAGACCGAACTTC
S382A-F	Site-directed mutagenesis	ATCCAATGCATCCACATTTGGCTCAAGACCCATTAA CTGGACAAACTATACTTTTAGGTC
S382A-R	Site-directed mutagenesis	TCCAGTTAATGGGTCTTGAGCCAAATGTGGATGCA TTGGATGATGGAGATGACTGCCATC
SBE1-ChIP-F	ChIP-qPCR	AGCTTCTGTGAATGAGGATGAAGAA
SBE1-ChIP-R	ChIP-qPCR	GTTGTTTACAGATCACCTGTGGACA
siHNF4G#1	Knockdown	5'-GCAGCAUUCGUAAGAGUCATT-3'
siHNF4G#2	Knockdown	5'-GAACGUGACAGAAUAAGCATT-3'
siSMAD4#1	Knockdown	5'-GGUGGAGAGAGUGAAACAUTT-3'
siSMAD4#2	Knockdown	5'-CCAGCAUCCACCAAGUAAUTT-3'
siPRKAA1#1	Knockdown	5'-GAGGAGAGCUAUUUGAUUATT-3'
siPRKAA1#2	Knockdown	5'-GCGUGUACGAAGGAAGAAUTT-3'
sgHNF4G#1	Knockout	5'-GCACTGTTGAGAGCTCACGC-3'
sgHNF4G#2	Knockout	5'-TCGGCAATGTGTTGTTGACA-3'
